# 3D Generative Model Latent Disentanglement via Local Eigenprojection

**DOI:** 10.1111/cgf.14793

**Published:** 2023-04-04

**Authors:** Simone Foti, Bongjin Koo, Danail Stoyanov, Matthew J. Clarkson

**Affiliations:** ^1^ University College London London UK; ^2^ University of California, Santa Barbara Santa Barbara USA

**Keywords:** disentanglement, generative adversarial networks, geometric deep learning, variational autoencoder

## Abstract

Designing realistic digital humans is extremely complex. Most data‐driven generative models used to simplify the creation of their underlying geometric shape do not offer control over the generation of local shape attributes. In this paper, we overcome this limitation by introducing a novel loss function grounded in spectral geometry and applicable to different neural‐network‐based generative models of 3D head and body meshes. Encouraging the latent variables of mesh variational autoencoders (VAEs) or generative adversarial networks (GANs) to follow the local eigenprojections of identity attributes, we improve latent disentanglement and properly decouple the attribute creation. Experimental results show that our local eigenprojection disentangled (LED) models not only offer improved disentanglement with respect to the state‐of‐the‐art, but also maintain good generation capabilities with training times comparable to the vanilla implementations of the models. Our code and pre‐trained models are available at github.com/simofoti/LocalEigenprojDisentangled.

## Graphical abstract

This work introduces a new loss function grounded in spectral geometry and applicable to different neural‐network‐based generative models of 3D head and body meshes. A model trained with our local eigenprojection loss (1), can be used to generate and edit human shapes by directly manipulating the latent variables (2).

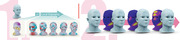

## 1 Introduction

In recent years digital humans have become central elements not only in the movie and video game production, but also in augmented and virtual reality applications. With a growing interest in the metaverse, simplified creation processes of diverse digital humans will become increasingly important. These processes will benefit experienced artists and, more importantly, will democratize the character generation process by allowing users with no artistic skills to easily create their unique avatars. Since digitally sculpting just the geometric shape of the head of a character can easily require a highly skilled digital artist weeks to months of work [GFZ*20], many semi‐automated avatar design tools have been developed. Even though simpler and faster to use, they inherit the intrinsic constraints of their underlying generative models [FKSC22]. Usually based upon blendshapes [LMR*15, OBB20, TDlTM11], principal component analysis (PCA) [BV99, PWP*19, LBB*17], variational autoencoders (VAEs) [RBSB18, GCBZ19, AATJD19, CNH*20], or generative adversarial networks (GANs) [CBZ*19, GLP*20, LBZ*20, ABWB19], these models are either limited in expressivity or they cannot control the creation of local attributes. Considering that deep‐learning‐based approaches, such as VAEs and GANs, offer superior representation capabilities with a reduced number of parameters and that they can be trained to encourage disentanglement, we focus our study on these models.

By definition [BCV13, HMP*17, KM18], with a disentangled latent representation, changes in one latent variable affect only one factor of variation while being invariant to changes in other factors. This is a desirable property to offer control over the generation of local shape attributes. However, latent disentanglement remains an open problem for generative models of 3D shapes [AATJD19] despite being a widely researched topic in the deep learning community [HMP*17, KM18, KWKT15, EWJ*19, DXX*20, WYH*21, RL21]. Most research on latent disentanglement of generative models for the 3D shape of digital humans addresses the problem of disentangling the pose and expression of a subject from its identity [AATJD19, AATDJ23, CNH*20, ABWB19, ZYL*20, LYF*21, ZYHC22], but none of these works is able to provide disentanglement over the latent variables controlling the local attributes characterizing the identity. Some control over the generation of local attributes was achieved for generative models of 3D furniture by leveraging complex architectures with multiple encoders and decoders independently operating on different furniture parts [NW17, YML*20, RDC*21]. In contrast, [FKSC22] recently proposed a method to train a single VAE while enforcing disentanglement among sets of latent variables controlling the identity of a character. This approach allows their Swap Disentangled VAE (SD‐VAE) to learn a more disentangled, interpretable, and structured latent representation for 3D VAEs of bodies and heads. However, although [FKSC22] disentangles subsets of latent variables controlling local identity attributes, variables within each set can be entangled and not orthogonal. In addition, their curated mini‐batching procedure based on attribute swapping is applicable only to autoencoder‐based architectures and it significantly increases the training duration. In this work, we aim at overcoming these limitations by leveraging spectral geometry to achieve disentanglement without curating the mini‐batching. In particular, we encourage the latent representation of a mesh to equal the most significant local eigenprojections of signed distances from the mean shape of the training data. Since the eigenprojections are computed using the eigenvectors of combinatorial Laplacian operators, we require meshes to be in dense point correspondence and to share the same topology. This is a standard requirement for most of the traditional [BV99, BRZ*16, DPSD20, GFZ*20, LMR*15, OBB20, PWP*19, PVS*21] and neural‐network‐based [FKD*20, FKSC22, GCBZ19, RBSB18, ZWL*20, YLY*20] generative models, which not only simplifies the shape generation process, but also the definition of other digital humans' properties that will be automatically shared by all the generated meshes (e.g. UV maps, landmarks, and animation rigs).

**Figure 1:**
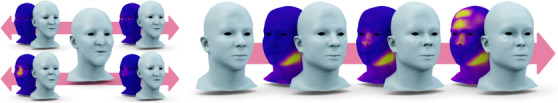
Shape generation and editing of two subjects randomly generated with LED‐VAE, which is one of the proposed local eigenprojection disentangled models. *Left*: effects caused on the generated shapes by traversing two arbitrary latent variables controlling the eyes and nose of the first random subject. *Right*: example of shape editing performed manipulating the latent variables controlling jaw, nose, and forehead of the second subject. The latent manipulations are performed with a GUI that allows the manual modification of the latent variables, but random per‐attribute modifications can also be performed. The edited shapes are always paired with their corresponding displacement map highlighting the shape differences from the initial model.

To summarize, the key contribution of this work is the introduction of a novel local eigenprojection loss, which is able to improve latent disentanglement among variables controlling the generation of local shape attributes contributing to the characterization of the identity of digital humans. Our method improves over SD‐VAE by enforcing orthogonality between latent variables and avoiding the curated mini‐batching procedure, thus significantly reducing the training times. In addition, we demonstrate the flexibility and disentanglement capabilities of our method on both VAEs and GANs.

## 2 Related Work

### 2.1 Generative models

Blendshapes are still widely adopted for character animation or as consumer‐level avatar design tools because, by linearly interpolating between a predefined set of artistically created shapes, the blend‐weights can be easily interpreted [LAR*14]. However, to compensate for the limited flexibility and diversity of these models, large amounts of shapes are required. This makes the models very large and only a limited number of shapes can be used in most practical applications. An alternative approach capable of offering more flexibility is to build models relying on principal component analysis (PCA) [BV99, EST*20]. These data‐driven models are able to generate shapes as linear combinations of the training data, but the variables controlling the output shapes are related to statistical properties of the training data and are difficult to interpret. In recent years, PCA‐based models have been created from large number of subjects. For example, lsfm [BRZ*16] and lyhm [DPSD20] were built collecting scans from 10,000 faces and 1212 heads, respectively. The two models were later combined in uhm [PWP*19], which was subsequently enriched with additional models for ears, eyes, teeth, tongue, and the inner‐mouth [PVS*21]. Also, [GFZ*20] combined multiple PCA models, but they were controlling different head regions and an anatomically constrained optimization was used to combine their outputs and thus create an interactive head sculpting tool. PCA‐based models of the body were also combined with blendshapes in smpl [LMR*15] and star [OBB20], which were trained with 3800 and 14,000 body scans respectively. PCA‐based models generally trade the amount of fine details they can represent with their size. The advent of geometric deep learning techniques brought a new set of operators making possible the creation of neural network architectures capable of processing 3D data such as point‐clouds and meshes. [RBSB18] introduced the first VAE for the generation of head meshes. In its comparison against PCA, the VAE model used significantly fewer parameters and exhibited superior performances in generalization, interpolation, and reconstruction. This pioneering work was followed by many other autoencoders which differed from one another mostly by their application domain and the mesh operators used in their architecture [LBBM18, FKD*20, YFST18, ZWL*20, GCBZ19, DS19, TZY*22, BBP*19]. These mesh operators were used also for generative models based on GAN architectures [OBD*21, CBZ*19], but they appear to be less frequent than their VAE counterparts. Most GAN architectures operate in the image domain by representing 3D shapes in a UV space [MPN*20, LBZ*20].

### 2.2 Latent disentanglement

Most research on latent disentanglement is performed on generative models of images [KSB18, KM18, KWKT15, EWJ*19, DXX*20, RL21, WYH*21]. The β‐VAE [HMP*17] is probably the simplest model used to improve disentanglement in a VAE. Other simple methods that leverage statistical properties and do not require supervision over the generative factors are for instance the DIP‐VAEs [KSB18] and the FactorVAE [KM18]. All methods above were re‐implemented to operate on meshes by [FKSC22], but they did not report good levels of disentanglement with respect to the identity attributes. In the 3D realm, there are currently two prominent streams of research: the one disentangling the identity from the pose or expression of digital humans [AATJD19, AATDJ23, CNH*20, ZYL*20, ZBPM20, TSL21, JWCZ19, HHS*21, OFD*22], and the stream attempting to disentangle parts of man‐made objects [YML*20, NW17, LLW22, RDC*21]. In both cases, the proposed solutions require complex architectures. In addition, in the former category, current state‐of‐the‐art methods do not attempt to disentangle identity attributes. The latter category appears better suited for this purpose, but the type of generated shapes is substantially different because the generation of object parts needs to consider intrinsic hierarchical relationships, and surface discontinuities are not a problem. More similar to ours, is the method recently proposed by [FKSC22], where the latent representation of a mesh convolutional VAE is disentangled by curating the mini‐batching procedure and introducing an additional loss. In particular, swapping shape attributes between the input meshes of every mini‐batch, it is possible to know which of them share the same attribute and which share all the others. This knowledge is harnessed by a contrastive‐like latent consistency loss that encourages subsets of latent variables from different meshes in the mini‐batch to assume the same similarities and differences of the shapes created with the attribute swapping. This disentangles subsets of latent variables which become responsible for the generation of different body and head attributes. We adopt the same network architecture, dataset, and attribute segmentation of SD‐VAE. This choice is arbitrary and simplifies comparisons between the two methods, which differ only in their disentanglement technique.

Like VAEs, the research on GANs comes mostly from the imaging domain, where good levels of control over the generation process were recently made possible. Most of these models leverage segmentation maps [HMWL22, LLWL20, LKL*21], additional attribute classifiers [HZK*19, SBKM21], text prompts [RKH*21], or manipulate the latent codes and the parameter space of the pre‐trained model to achieve the desired results [KAL*21, HHLP20, SYTZ22, LKL*21]. We argue that while the first two approaches require more inputs and supervision than our method, the last two offer less editing flexibility. In fact, describing the shape of human parts is a difficult task that would ultimately limit the diversity of the generated shapes, while the post‐training manipulation may limit the exploration of some latent regions. Only a few methods explicitly seek disentanglement during training [AW20, VB20] like ours. However, [AW20] is specifically designed for grid‐structured data, like images, and [VB20] still requires a pre‐trained GAN and two additional networks for disentanglement. In the 3D shapes domain, GAN disentanglement is still researched to control subject poses and expressions [CTS*21, OBD*21] or object parts [LLHF21]. However, they suffer the same problems described for 3D VAEs: they have complex architectures and do not have control over the generation of local identity attributes.

### 2.3 Spectral geometry

Spectral mesh processing has played an essential role in shape indexing, sequencing, segmentation, parametrization, correspondence, and compression [ZVKD10]. Spectral methods usually leverage the properties of the eigenstructures of operators such as the mesh Laplacian. Even though there is no unique definition for this linear operator, it can be classified either as geometric or combinatorial. Geometric Laplacians are a discretization of the continuous Laplace‐Beltrami operator [Cha84] and, as their name suggests, they encode geometric information. Their eigenvalues are robust to changes in mesh connectivity and are often used as shape descriptors[RWP06, GYP14]. Since they are isometry‐invariant, they are used also in VAEs for identity and pose disentanglement [AATJD19, AATDJ23]. However, being geometry dependant, the Laplace‐Beltrami operator and its eigendecomposition have to be precomputed for every mesh in the dataset. On the other hand, combinatorial Laplacians treat a mesh as a graph and are entirely defined by the mesh topology. For these operators, the eigenvectors can be considered as Fourier bases and the eigenprojections are equivalent to a Fourier transformation [SNF*13] whose result is often used as a shape descriptor. If all shapes in a dataset share the same topology, the combinatorial Laplacian and its eigendecomposition need to be computed only once. For this reason, multiple graph and mesh convolutions [BZSL13, DBV16] as well as some data augmentation techniques [FKD*20] and smoothing losses [FKSC22] are based on combinatorial Laplacian formulations.

## 3 Method

The proposed method introduces a novel loss to improve latent disentanglement in generative models of 3D human shapes. After defining the adopted shape representation, we introduce our local eigenprojection loss, followed by the two generative models on which it was tested: a VAE and two flavours of GANs.

### 3.1 Shape representation

We represent 3D shapes as manifold triangle meshes with a fixed topology. By fixing the topology, all meshes M={X,E,F} share the same edges $E\in N^{\varepsilon \times 2}$ and faces $F\in N^{\Gamma \times 3}$. Therefore, they differ from one another only for the position of their vertices $X\in R^{N\times 3}$, which are assumed to be consistently aligned, scaled, and with point‐wise correspondences across shapes.

### 3.2 Local eigenprojection loss

**Figure 2:**
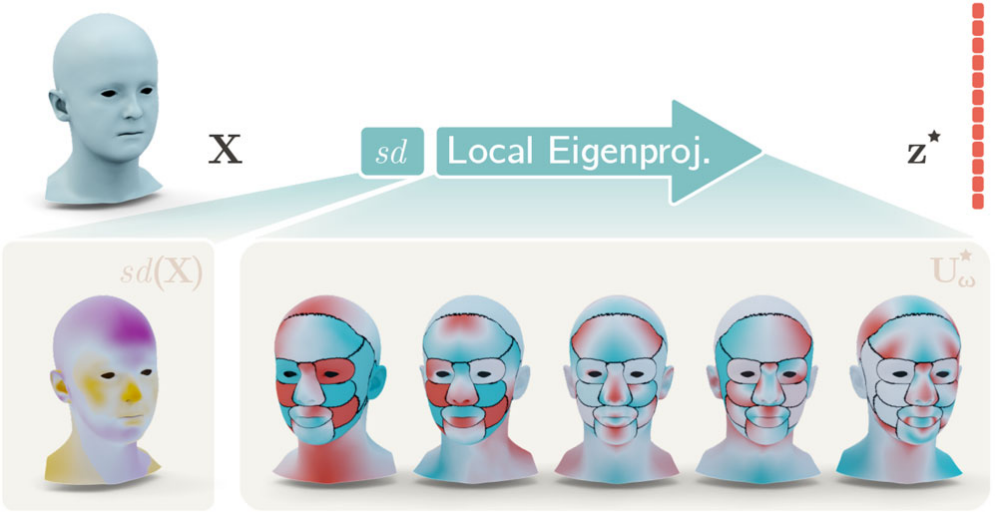
Schematic representation of the local eigenprojection, the operation at the core of our local eigenprojection loss. The signed distance between a given mesh X and a mean shape template is computed as sd(X). sd(X) is locally eigenprojected into a vector $z^{★}$ where each subset of variables is a spectral descriptor of a shape attribute. The projection is performed by matrix‐multiplying the signed distance by $U_{\omega }^{★}$, the highest‐variance eigenvectors of each shape attribute ω. The heads in the bottom part of the figure represent one‐dimensional vectors whose values are mapped with diverging colour maps on the mean shape head. On the heads corresponding to the columns of $U_{\omega }^{★}$, the black seams mark the different attributes that we seek to control during the generation procedure.

We define *F* arbitrary attributes on a mesh template by manually colouring anatomical regions on its vertices. Thanks to the assumption of our shape representation, the segmentation of the template mesh can be consistently transferred to all the other meshes without manually segmenting them. Mesh vertices can be then grouped per‐attribute such that $X={\left\{X_{\omega }\right\}}_{\omega =1}^{F}$. Seeking to train generative models capable of controlling the position of vertices corresponding to each shape attribute $X_{\omega }$ through a predefined set of latent variables, we evenly split the latent representation z in *F* subsets of size κ, such that $z={\left\{z_{\omega }\right\}}_{\omega =1}^{F}$ and each $z_{\omega }$ controls its corresponding $X_{\omega }$. To establish and enforce a direct relationship between each $X_{\omega }$ and $z_{\omega }$ we rely on spectral geometry and compute low‐dimensional local shape descriptors in the spectral domain. We start by computing the Kirchoff graph Laplacian corresponding to each shape attribute as: $K_{\omega }=D_{\omega }-A_{\omega }$, where $A_{\omega }\in N^{N_{\omega }\times N_{\omega }}$ is the adjacency matrix of attribute ω, $D_{\omega }\in R^{N_{\omega }\times N_{\omega }}$ its diagonal degree matrix, and $N_{\omega }$ the number of its vertices. Values on the diagonal of $D_{\omega }$ are computed as $D_{aa}=\sum_{b}A_{ab}$. The Kirchoff Laplacian is a real symmetric positive semidefinite matrix that can be eigendecomposed as $K_{\omega }=U_{\omega }\Lambda_{\omega }U_{\omega }^{T}$. The columns of $U_{\omega }\in R^{N_{\omega }\times K}$ are a set of *K* orthonormal eigenvectors known as the graph Fourier modes and can be used to transform any discrete function defined on the mesh vertices into the spectral domain. The signal most commonly transformed is the mesh geometry, which is the signal specifying the vertex coordinates. However, the local eigenprojection ${\tilde{X}}_{\omega }=U_{\omega }^{T}X_{\omega }$ would result in a matrix of size K×3 containing the spectral representations of the 3 spatial coordinates. Instead of flattening ${\tilde{X}}_{\omega }$ to make it compatible with the shape of the latent representation, we define and project a one‐dimensional signal: the signed distance between the vertices of a mesh and the per‐vertex mean of the training set M (see Figure 2). We have:

(1) $$sd\left(X\right)={\gamma ∥X-M∥}_{2}\;\text{with}\;\gamma =\text{sign}\left(〈X-M,N〉\right),$$

where ⟨·,·⟩ is the inner product, and N are the vertex normals referred to the mesh template with vertex positions M. If X was standardized by subtracting M and dividing by the per‐vertex standard deviation of the training set ***Σ***, being ⊙ the Hadamard product, Equation 1 can be rewritten as:

(2) $$sd\left(X\right)={\gamma ∥X⊙\Sigma ∥}_{2}\;\text{with}\;\gamma =\text{sign}\left(〈X⊙\Sigma ,N〉\right).$$

We assume that not all eigenprojections are equally significant when representing shapes. Therefore, for each attribute ω, we eigenproject all the local signed distances $sd(X_{\omega })$ computed over the training set, and identify the κ (with κ≪K) spectral components with the highest variance. While these spectral components are responsible for most shape variations, the small shape differences represented by other components can be easily learned by the neural‐network‐based generative model. After eigenprojecting the entire training set, we select the Fourier modes $U_{\omega }^{★}\in R^{N_{\omega }\times \kappa }$ associated with the highest variance eigenprojections (Figure 2) and use them to compute the eigenprojection loss. During this preprocessing step, we also compute the mean and standard deviation of the highest variance local eigenrpojections, which we denote by $m_{\omega }^{★}$ and $s_{\omega }^{★}$, respectively. We thus define the local eigenprojection loss as:

(3) $$L_{\mathrm{LE}}\left(X,z\right)=\frac{1}{F\kappa }\sum_{\omega =1}^{F}∥z_{\omega }-\frac{{\left(U_{\omega }^{★}\right)}^{T}sd\left(X_{\omega }\right)-m_{\omega }^{★}}{s_{\omega }^{★}}{∥}_{1}$$

Note that combinatorial Laplacian operators are determined exclusively by the mesh topology. Since the topology is fixed across the dataset, the Laplacians and their eigendecompositions can be computed only once. Therefore, the local eigenprojection can be quickly determined by matrix‐multiplying signed distances by the precomputed $U_{\omega }^{★}$. Also, if the Laplace‐Beltrami operator was used in place of the Kirchoff graph Laplacian, the eigendecomposition would need to be computed for every mesh. Not only this would significantly increase the training duration, but backpropagating through the eigendecomposition would be more complex as this would introduce numerical instabilities [WDH*19]. Alternatively, an approach similar to [MRC*21] should be followed.

### 3.3 Mesh variational autoencoder

Like traditional VAEs [KW14], our 3D‐VAE is also built as a probabilistic encoder‐decoder pair parameterized by two separate neural networks. The probabilistic encoder is defined as a variational distribution q(z|X) that approximates the intractable model posterior. It predicts the mean ***μ*** and standard deviation ***σ*** of a Gaussian distribution over the possible z values from which X could have been generated. The probabilistic decoder p(X|z) describes the distribution of the decoded variable given the encoded one. During the generation process, a latent vector z is sampled from a Gaussian prior distribution p(z)=N(z;0,I) and an output shape is generated by the probabilistic decoder. Since the decoder is used as a generative model, it is also referred to as generator. Following this convention, we define our architecture as a pair of non‐linear functions {E,G}, where E:X→Z maps from the vertex embedding domain X to the latent distribution domain Z, and G:Z→X vice versa. Since traditional convolutional operators are not compatible with the non‐Euclidean nature of meshes, we build both networks as in [FKSC22], using the simple yet efficient spiral convolutions [GCBZ19] and sparse matrix multiplications with transformation matrices obtained with quadric sampling [GCBZ19, RBSB18] (see Supplementary Materials for more details).

As in [FKSC22], the 3D‐VAE is trained minimizing $L_{\mathrm{VAE}}=L_{R}\left(X,X^{\prime }\right)+\alpha L_{L}\left(X^{\prime }\right)+\beta L_{\mathrm{KL}}\left(\mu ,\sigma \right)$. While α and β are weighting constants, $L_{R}$ is the reconstruction loss, $L_{L}$ is a Laplacian regularizer, and $L_{\mathrm{KL}}$ is a Kullback–Leibler (KL) divergence. In auto‐encoder parlance, the reconstruction loss $L_{R}\left(X,X^{\prime }\right)=\frac{1}{N}{∥X^{\prime }-X∥}_{F}^{2}$ encourages the output of the VAE to be as close as possible to its input by computing the squared Frobenius norm between $X^{\prime }=G\left(E\left(X\right)\right)$ and X. The KL divergence can be considered as a regularization term that pushes the variational distribution q(z|X) towards the prior distribution p(z). Since both prior and posterior are assumed to be Gaussian $L_{\mathrm{KL}}\left(\mu ,\sigma \right)=\sigma^{2}+\mu^{2}-\log\left(\sigma \right)-1$. The Laplacian loss $L_{L}\left(X^{\prime }\right)=\frac{1}{N}{∥{\mathrm{TX}}^{\prime }∥}_{F}^{2}$ is a smoothing term computed on the output vertices $X^{\prime }$ and based on the Tutte Laplacian $T=D^{-1}K=I-D^{-1}A$, where A, D, and K are the adjacency, diagonal degree, and Kirchoff Laplacian introduced in the previous paragraph and computed on the entire mesh rather than on shape attributes.

Latent disentanglement is enforced by separately applying the local eigenprojection loss to the encoder and generator. We thus define the total loss as:

(4) $$\begin{array}{ccc} L & = & L_{R}\left(X,X^{\prime }\right)+\alpha L_{L}\left(X^{\prime }\right)+\beta L_{\mathrm{KL}}\left(\mu ,\sigma \right)\\ & & +\;\eta_{1}L_{\mathrm{LE}}\left(X,\mu \right)+\eta_{2}L_{\mathrm{LE}}\left(X^{\prime },\mu \right), \end{array}$$

where η_1_ and η_2_ are two scalar weights balancing the contributions of the two local eigenprojection losses. Note that $L_{\mathrm{LE}}\left(X,\mu \right)$ is backpropagated only through *E*. This term pushes the predicted ***μ*** towards the standardized local eigenprojections of the input, while the KL divergence attempts to evenly distribute the encodings around the centre of the latent space. Similarly, $L_{\mathrm{LE}}\left(X^{\prime },\mu \right)$ is backpropagated only through *G* and it enforces the output attributes to have an eigenprojection compatible with the predicted mean.

### 3.4 Mesh generative adversarial networks

We propose two flavours of 3D Generative Adversarial Networks: one based on Least Squares GAN (LSGAN) [MLX*17] and one on Wasserstein GAN (WGAN) [ACB17]. Like VAEs, GANs also rely on a pair of neural networks: a generator‐discriminator pair {G,D} in LSGAN and a generator‐critic {G,C} pair in WGAN. The architecture of the generators is the same as the one adopted in the generator of the 3D‐VAE. The architectures of *D* and *C* are similar to *E*, but with minor differences in the last layers (see Supplementary Materials). Nevertheless, all networks are built with the same mesh operators of our 3D‐VAE and [FKSC22, GCBZ19].

In the LSGAN implementation, *G* samples an input latent representation from a Gaussian distribution p(z)=N(z;0,I) and maps it to the shape space as $G\left(z\right)=X^{\prime }$. While it tries to learn a distribution over generated shapes, the discriminator operates as a classifier trying to distinguish generated shapes $X^{\prime }$ from real shapes X. Using a binary coding scheme for the labels of real and generated samples, we can write the losses of *G* and *D* respectively as $L_{\mathrm{LSGAN}}^{G}=\frac{1}{2}E_{z∼p(z)}\left[{\left(D\left(G\left(z\right)\right)-1\right)}^{2}\right]$ and $L_{\mathrm{LSGAN}}^{D}=\frac{1}{2}E_{X∼p(X)}\left[{\left(D\left(X\right)-1\right)}^{2}\right]+\frac{1}{2}E_{z∼p(z)}\left[D{\left(G\left(z\right)\right)}^{2}\right]$. We also add the Laplacian regularization term $L_{L}\left(X^{\prime }\right)$ to smooth the generated outputs. When seeking disentanglement, we train the discriminator by minimizing $L_{\mathrm{LSGAN}}^{D}$ and the generator by minimizing the following:

(5) $$L_{\mathrm{LS}}^{G}=L_{\mathrm{LSGAN}}^{G}+\alpha L_{L}\left(X^{\prime }\right)+\eta L_{\mathrm{LE}}\left(X^{\prime },z\right).$$

In WGAN, *G* still tries to learn a distribution over generated shapes, but its critic network *C*, instead of classifying real and generated shapes, learns a Wasserstein distance and outputs scalar scores that can be interpreted as measures of realism for the shapes it processes. The WGAN losses for *G* and *C* are $L_{\mathrm{WGAN}}^{G}=-E_{z∼p(z)}[D\left(G\left(z\right)\right]$ and $L_{\mathrm{WGAN}}^{C}=E_{z∼p(z)}\left[D\left(G\left(z\right)\right)\right]-E_{X∼p(X)}\left[D\left(X\right)\right]$ respectively. Similarly to the LSGAN implementation, when enforcing disentanglement, the critic is trained minimizing $L_{\mathrm{WGAN}}^{C}$, while the generator minimizing:

(6) $$L_{W}^{G}=L_{\mathrm{WGAN}}^{G}+\alpha L_{L}\left(X^{\prime }\right)+\eta L_{\mathrm{LE}}\left(X^{\prime },z\right).$$

Note that to make *C* a 1‐Lipschitz function, and thus satisfies the Wasserstein distance computation requirements, *C* weights are clipped to the range [−c,c].

## 4 Experiments

### 4.1 Datasets

Since our main objective is to train a generative model capable of generating different identities, we require datasets containing a sufficient number of subjects in a neutral expression (pose). Most open source datasets for 3D shapes of faces, heads, bodies, or animals (e.g. mpi‐Dyna [PMRMB15], smpl [LMR*15], surreal [VRM*17], Coma [RBSB18], smal [ZKJB17], etc.) focus on capturing different expressions or poses and are not suitable for identity disentanglement. For comparison, we rely on the 10,000 meshes – with neutral expression and pose – generated in [FKSC22] using two linear models that were built using a large number of subjects: uhm [PWP*19] and star [OBB20] (Section 2.1). We also use the same data split with 90% of the data for training, 5% for validation, and 5% for testing. Since these data are generated from PCA‐based models, we also train our models on real data from the lyhm dataset [DPSD20] registered on the flame [LBB*17] template. In addition, even though it is beyond the scope of this work, we attempt to achieve disentanglement through local eigenprojection also on Coma [RBSB18], a dataset mostly known for its wide variety of expressions. All models and datasets are released for non‐commercial scientific research purposes.

### 4.2 Local eigenprojection distributions

**Figure 3:**
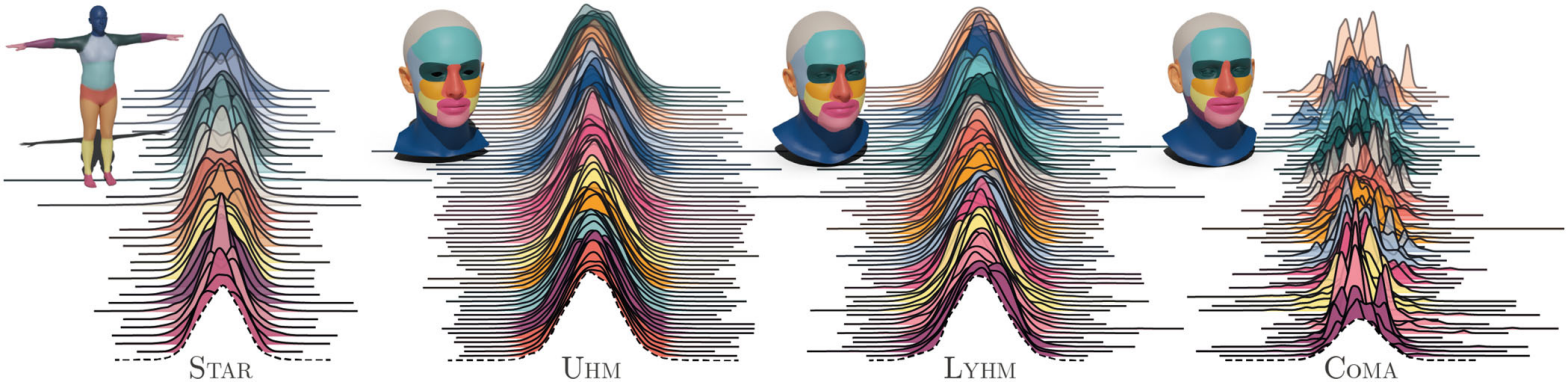
Local eigenprojection distributions. All training meshes are locally eigenprojected to observe the distributions of the elements in the resulting vectors. Distributions are colour‐coded according to the shape attribute they are referred to. The segmentation of the shape attributes displayed next to the distributions is rendered on the mean shape templates of the corresponding dataset. The dashed distributions, which are obtained sampling a Gaussian, are reported for comparison.

We observe that the eigenprojections are normally distributed for datasets with neutral poses or expressions (Figure 3). By standardizing the eigenprojections in Equation (3) we ensure their mean and standard deviation to be 0 and 1, respectively. Since we enforce a direct relation between the local eigenprojections and the latent representations, this is a desirable property that allows us to generate meaningful shapes by sampling latent vectors from a normal distribution. In order to explain why this property holds for datasets with neutral poses and expressions, we need to hypothesize that shapes follow a Gaussian distribution. This is a reasonable hypothesis for datasets generated from PCA‐based models, such as those obtained from uhm and star, because vertex positions are computed as linear combinations of generative coefficients sampled from a Gaussian. However, following the maximum entropy explanation [Lyo14], it is also reasonable to assume that shapes in dataset obtained capturing real people (like lyhm), are normally distributed. [Lyo14] argues that although the Central Limit Theorem is the standard explanation of why many things are normally distributed, the conditions to apply the theorem are usually not met or they cannot be verified. We assume that, like people's height, also body and head shapes are largely determined by genetics and partially by environment and epigenetic effects. The selection pressure determines an ideal shape with some variability to hedge against fluctuating circumstances in the environment. This amounts to fixing the mean, and an upper bound on the variance. Apart from that, the population will naturally tend to a state of maximal disorder (i.e. maximum entropy). Therefore, according to the maximum entropy explanation, human shapes are normally distributed because the distribution maximizing entropy subject to those constraints is a normal distribution. If the shapes are normally distributed, we can consider also vertex positions consistently sampled on the shape surfaces to follow each a different Gaussian distribution centred at the corresponding vertex coordinates on the mean shape. Considering that the signed distance and the local eigenprojection are both linear operations, they preserve normality, and for this reason also the local eigenprojections are normal. Note that expressions are subject‐specific deformations with highly non‐linear behaviour [CBGB20]. There is no guarantee that these transformations preserve the normality of the shape distribution. Therefore, datasets containing expressions, such as Coma, may not satisfy the normality assumption. In fact, we observe that the standardized eigenprojections have more complex distributions which appear to be mixture of Gaussians (see Figure 3). Intuitively, each Gaussian in the mixtures could be related to a different subset of expressions.

### 4.3 Comparison with other methods

We compare our local eigenprojection disentangled (LED) methods against their vanilla implementations and against the only state‐of‐the‐art method providing control over the generation of local shape attributes: the swap disentangled VAE (SD‐VAE) proposed in [FKSC22]. The authors compared their SD‐VAE with other VAEs for latent disentanglement. Among their implementation of DIP‐VAE‐I, DIP‐VAE‐II, and FactorVAE, the first one appeared to be the best performing. Therefore, we report results for DIP‐VAE‐I. For a fair comparison, all methods were trained on the same dataset (uhm) using the same batch size and the same number of epochs. In addition, they share the same architecture with minor modifications for the GAN implementations (see Supplementary Materials). The SD‐VAE implementation, as well as the evaluation code and the benchmark methods, are made publicly available at github.com/simofoti/3DVAE‐SwapDisentangled. All models were trained on a single Nvidia Quadro P5000, which was used for approximately 18 GPU days in order to complete all the experiments.

The reconstruction errors reported in Table 1 are computed as the mean per‐vertex L2 distance between input and output vertex positions. This metric is computed on the test set and applies only to VAEs. We report the generation capabilities of all models in terms of diversity, JSD, MMD, COV, and 1‐NNA. The diversity is computed as the average of the mean per‐vertex distances among pairs of randomly generated meshes. The Jensen‐Shannon Divergence (JSD) [ADMG18] evaluates the KL distances between the marginal point distributions of real and generated shapes. The coverage (COV) [ADMG18] measures the fraction of meshes matched to at least one mesh from the reference set. The minimum matching distance (MMD) [ADMG18] complements the coverage by averaging the distance between each mesh in the test set and its nearest neighbour among the generated ones. The 1‐nearest neighbour accuracy (1‐NNA) is a measure of similarity between shape distributions that evaluates the leave‐one‐out accuracy of a 1‐NN classifier. In its original formulation [YHH*19], it expects values converging to 50%. However, following [FKSC22], in Table 1 we report absolute differences between the original score and the 50% target value. All the generation capability metrics can be computed either with the Chamfer or the Earth Mover distance. Since we did not observe significant discrepancies between the metrics computed with these two distances, we arbitrarily report results obtained with the Chamfer distance.

**Table 1:** Quantitative comparison between our model and other state‐of‐the‐art methods. All methods were trained on uhm [PWP*19]. Diversity, JSD, MMD, COV, and 1‐NNA evaluate the generation capabilities of the models, while VP evaluates latent disentanglement. The different metrics are computed as detailed in Section 4.3. Note that the training time does not consider the initialization time.

Method	Mean Rec. (↓)	Diversity (↑)	JSD (↓)	MMD (↓)	COV (%, ↑)	1‐NNA (Δ%, ↓)	VP (%, ↑)	Training Time (↓)
VAE	0.61	4.23	4.89	1.53	65.49	1.17	63.73	1h:46m
LSGAN	—	6.12	1.14	1.65	43.41	22.04	46.83	2h:23m
WGAN	—	4.04	22.75	1.36	57.94	23.98	71.07	2h:22m
DIP‐VAE‐I	4.65	4.74	5.32	1.24	55.57	4.31	35.60	1h:48m
SD‐VAE	0.73	4.23	4.30	1.56	65.67	0.50	79.75	7h:21m
LED‐VAE	1.46	5.30	2.27	1.73	49.83	15.80	80.75	2h:53m
LED‐LSGAN	—	6.38	2.09	2.03	43.41	17.23	79.75	2h:28m
LED‐WGAN	—	5.77	2.55	1.81	47.47	14.95	74.11	2h:28m

**Figure 4:**
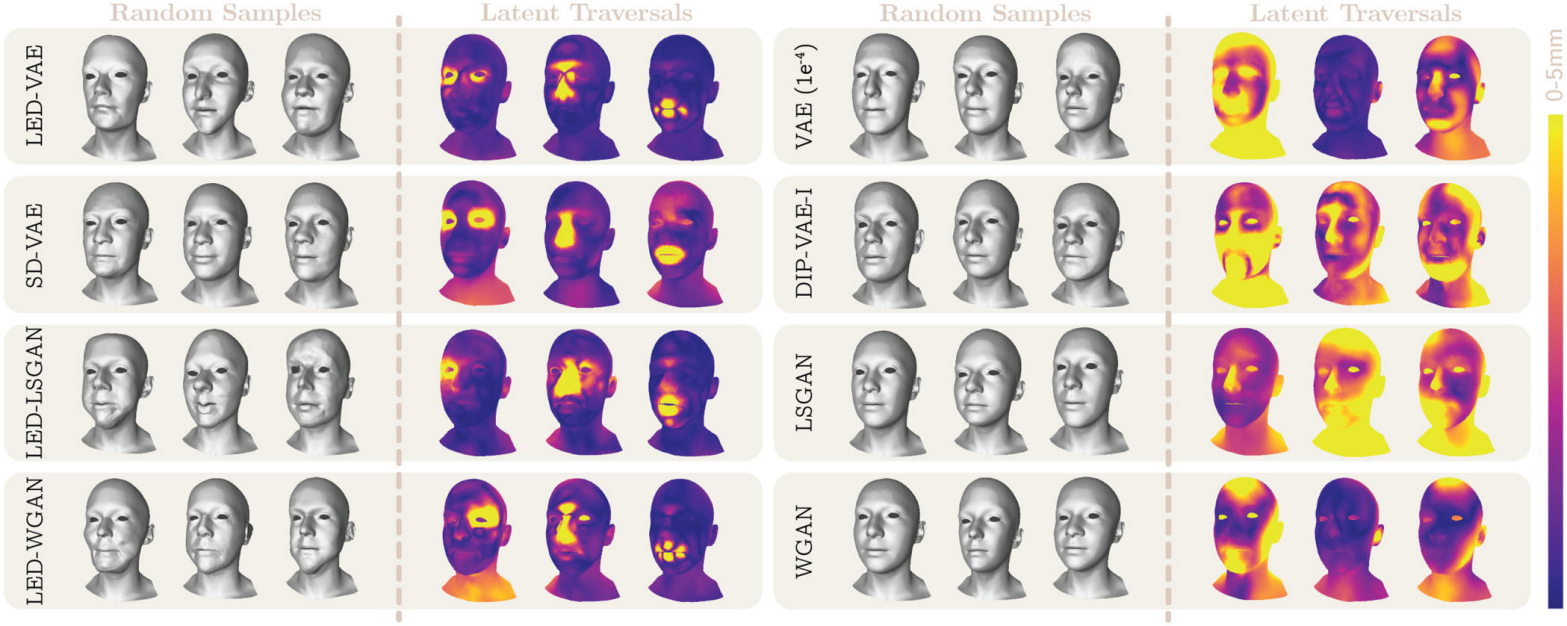
Random samples and vertex‐wise distances showing the effects of traversing three randomly selected latent variables (see Supplementary Materials to observe the effects for all the latent variables).

Observing Table 1 we notice that none of the models is consistently outperforming the others. GANs generally report better diversity scores than VAEs, but they are worse in terms of coverage and 1‐NNA. GANs were also more difficult to train and were prone to mode collapse. On the other hand, VAEs appeared stable and required significantly less hyperparameter tuning. The scores of our LED models were comparable with other methods, thus showing that our loss does not negatively affect the generation capabilities. However, LED models are consistently outperformed in terms of MMD, COV, and 1‐NNA. These metrics evaluate the quality of generated samples by comparing them to a reference set. Since comparisons are performed on the entire output shapes, we hypothesize that a shape with local identity attributes resembling each a different subject from the test set is more penalized than a shape whose attributes are plausibly obtained from a single subject. Note also that MMD, COV, and 1‐NNA appear to be inversely proportional to the diversity, suggesting that more diverse generated shapes are also less similar to shapes in the test set. LED‐models report higher diversity because attributes can be independently generated. This negatively affects MMD, COV, and 1‐NNA, but the randomly generated shapes are still plausible subjects (see Figure 4 and Supplementary Materials). Interestingly, SD‐VAE appears to be still capable of generating shapes with attributes resembling the same subject from the test set, but at the expense of diversity and latent disengagement (see Section 4.4).

LED‐LSGAN and LED‐WGAN train almost as quickly as the vanilla LSGAN and WGAN. Training LED‐VAE takes approximately 1 h more than its vanilla counterpart because the local eigenprojection loss is separately backpropagated through the encoder. However, since latent disentanglement is achieved without swapping shape attributes during mini‐batching, the training time of LED‐VAE is reduced by 61% with respect to SD‐VAE. Note that the additional initialization overhead of LED models (3.72 min) is negligible when compared to the significant training time reduction over SD‐VAE, which is the only model capable of achieving a satisfactory amount of latent disentanglement.

If we then qualitatively evaluate the random samples in Figure 4, we see that the quality of the meshes generated by LED‐LSGAN and LED‐WGAN is slightly worse than those from LED‐VAE. We attribute this behaviour to the –usually undesired– smoothness typically introduced by 3D VAE models. In this case, the VAE model itself acts as a regularizer that prevents the shape artefacts introduced by the local eigenprojection disentanglement. In addition, traversing the latent variables, we find that mesh defects tend to appear when latent variables approach values near ±3 (see Supplementary Material video). This might be a consequence of the reduced number of training data with local eigenprojections with these values (see Figure 3). Nonetheless, the problem can be easily mitigated with the truncation trick, thus sampling latent vectors from a Gaussian with standard deviation slightly smaller than one.

### 4.4 Evaluation of latent disentanglement

Latent disentanglement can be quantitatively evaluated on datasets with labelled data. However, such labels are not available for the disentanglement of shape attributes and traditional metrics such as Z‐Diff [HMP*17], SAP [KSB18], and Factor [KM18] scores cannot be used. Since the Variation Predictability (VP) disentanglement metric does not require labelled data and it has shown good correlation with the Factor score [ZXT20], we rely on this metric to quantify disentanglement across different models (see Table 1). The VP metric averages the test accuracies across multiple few‐shot training of a classifier. The classifier takes as input the difference between two shapes generated from two latent vectors differing in only one dimension and predicts the varied latent dimension. We implement the classifier network with the same architecture of our encoders, discriminators, and critiques. The network was trained for five epochs with a learning rate of $1e^{-4}$. As in [ZXT20], we set $\eta_{VP}=0.1$, $N_{VP}=10,000$ and $S_{VP}=3$.

**Figure 5:**
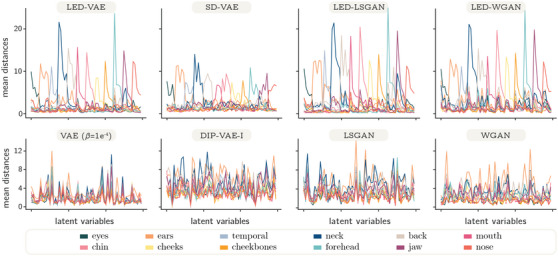
Effects of traversing each latent variable across different mesh attributes. For each latent variable (abscissas) we represent the per‐attribute mean distances computed after traversing the latent variable from its minimum to its maximum value. For each latent variable, we expect a high mean distance in one single attribute and low values for all the others.

**Figure 6:**
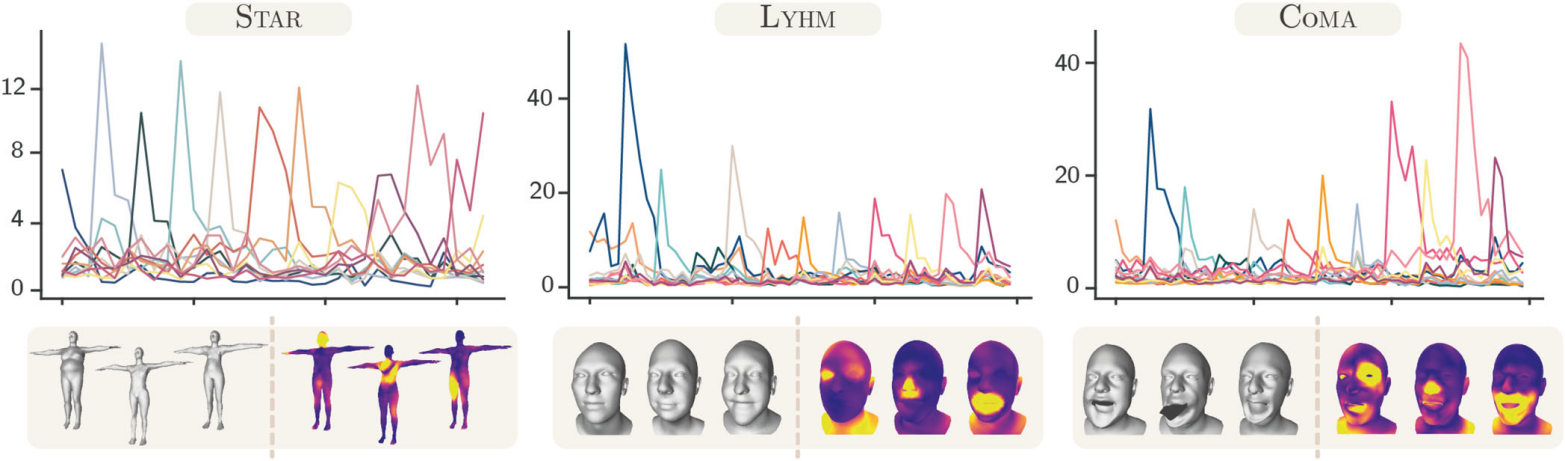
Results of LED‐VAE on other datasets. For each dataset are displayed the effects of traversing latent variables (uhm is reported in Figure 5), three random samples and three vertex‐wise distances highlighting the effects of traversing three latent variables (uhm is reported Figure 4). Mean distances are plotted following the colour coding depicted in Figure 3.

In addition, we qualitatively evaluate disentanglement as in [FKSC22] by observing the effects of traversing latent variables (Figure 1, *left*). For each latent variable, we compute the per‐vertex Euclidean distances between two meshes. After setting all latent variables to their mean value (0), the first mesh is generated setting a single latent to its minimum (−3) and the second mesh setting the same variable to its maximum (+3). The Euclidean distances can be either rendered on the mesh surface using colours proportional to the distances (Latent Traversals in Figure 4 and Figure 6), or plotted as their per‐attribute average distance (Figure 5 and Figure 6). When plotted, the average distances isolated to each attribute provide an intuitive way to assess disentanglement: good disentanglement is achieved when the traversal of a single variable determines high mean distances for one attribute and low mean distances for all the others. Observing Figure 4 and Figure 5, it is clear that the only state‐of‐the‐art method providing control over local shape attributes is SD‐VAE. Since the eigenvectors used in the local eigenprojection loss are orthogonal, we improve disentanglement over SD‐VAE. In fact, traversing latent variables of LED models determines finer changes within each attribute in the generated shapes. For instance, this can be appreciated by observing the eyes of the latent traversals in Figure 4, where left and right eyes are controlled by different variables in LED‐VAE, while by the same one in SD‐VAE (more examples are depicted in the Supplementary Materials). We also notice that the magnitude of the mean distances reported in Figure 5 for our LED models is bigger than SD‐VAE within attributes and comparable outside. This shows superior disentanglement and allows our models to generate shapes with more diverse attributes than SD‐VAE. Our model exhibits good disentanglement performances also on other datasets (Figure 6).

### 4.5 Direct manipulation

**Figure 7:**
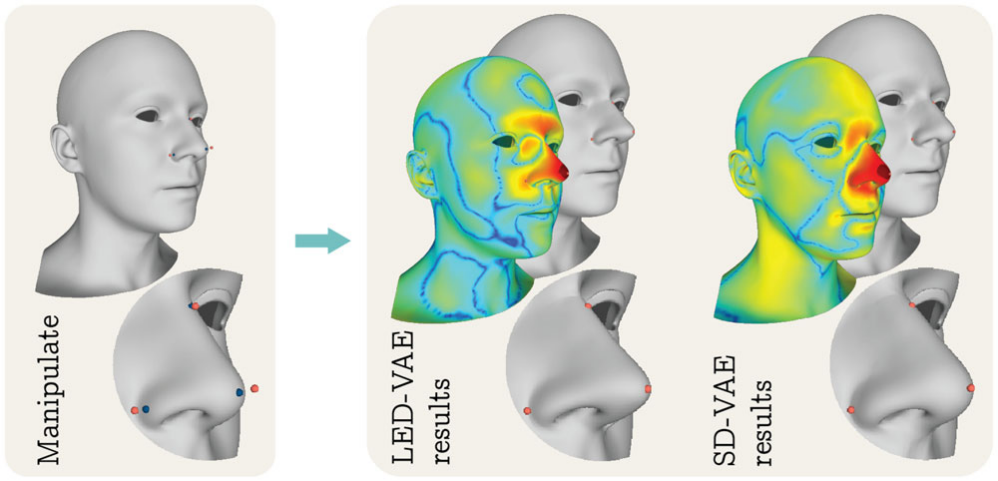
Direct manipulation. *Left*: the user manually selects an arbitrary number of vertices (blue) and specifies their desired position (red). *Right*: results of the direct manipulation optimization for LED‐VAE and SD‐VAE. For each method, the output shape, a close‐up of the manipulated attribute, and the rendering of the per‐vertex distances between the initial and manipulated shapes are reported. The colour‐map used to represent vertex distances is blue where distances are zero and red where they reach their maximum value.

Like SD‐VAE, also LED‐VAE can be used for the direct manipulation of the generated shapes. As in [FKSC22], the direct manipulation is performed by manually selecting Υ vertices on the mesh surface ($S\circ X^{\prime }=S\circ G\left(z\right)\in R^{\Upsilon \times 3}$) and by providing their desired location ($Y\in R^{\Upsilon \times 3}$). Then, $\min_{z_{\omega }}{∥S\circ G\left(z\right)-Y∥}_{2}^{2}$ is optimized with the adam optimizer for 50 iterations while maintaining a fixed learning rate of lr=0.1. Note that the optimization is performed only on the subset of latent variables $z_{\omega }$ controlling the local attribute corresponding to the selected vertices. If vertices from different attributes are selected, multiple optimizations are performed. As it can be observed in Figure 7, LED‐VAE is able to perform direct manipulations causing fewer shape changes than SD‐VAE in areas that should remain unchanged.

## 5 Conclusion

We introduced a new approach to train generative models with a more disentangled, interpretable and structured latent representation that significantly reduces the computational burden required by SD‐VAE. By establishing a correspondence between local eigenprojections and latent variables, generative models can better control the creation and modification of local identity attributes of human shapes (see Figure 1). Like the majority of state‐of‐the‐art methods, the main limitation of our model is the assumption on the training data, which need to be consistently aligned, in dense point correspondence, and with a fixed topology. Even though this is surely a limitation, as we mentioned in Section 1, this assumption can simplify the generation of other digital human's properties. Among the different LED models we proposed, we consider LED‐VAE to be the most promising. This model is simpler to train, requires less hyperparameter tuning, and generates higher‐quality meshes. We trained and tested this model also on other datasets, where it showed equivalent performances. Datasets with expressions have complex local eigenprojection distributions (Figure 3) which are more difficult to learn. In fact, random samples generated by LED‐VAE trained on Coma present mesh defects localized especially in areas where changes in expression introduce significant shape differences characterized by a highly non‐linear behaviour (e.g. the mouth region). Controlling the generation of different expressions was beyond the scope of this work and we aim at addressing the issue as future work. We proved that our loss can be easily used with both GANs and VAEs. Being efficient to compute and not requiring modifications to the mini‐batching procedure (like SD‐VAE), it could be leveraged also in more complex architectures for 3D reconstruction or pose and expression disentanglement. In the LED‐VAE the local eigenprojection loss is computed also on the encoder (see how this improves disentanglement in the ablation study provided with the supplementary materials). Having an encoder capable of providing a disentangled representation for different attributes could greatly benefit shape‐analysis research in plastic surgery [OvdLP*22] and in genetic applications [CRW*18]. Therefore, we believe that our method has the potential to benefit not only experienced digital artists but also democratize the creation of realistic avatars for the metaverse and find new applications in shape analysis. Since the generation of geometric shapes is only the first step towards the data‐driven generation of realistic digital humans, as future work, we will research more interpretable generative processes for expressions, poses, textures, materials, high‐frequency details, and hair.

## Supporting information

Video S1

Supporting Information

## References

[cgf14793-bib-0001] [AATDJ23] Aumentado‐Armstrong T. , Tsogkas S. , Dickinson S. , Jepson A. : Disentangling geometric deformation spaces in generative latent shape models. In International Journal of Computer Vision (2023).

[cgf14793-bib-0002] [AATJD19] Aumentado‐Armstrong T. , Tsogkas S. , Jepson A. , Dickinson S. : Geometric disentanglement for generative latent shape models. In Proceedings of the IEEE/CVF International Conference on Computer Vision . IEEE, Seoul, Korea (South) (2019), pp. 8181–8190.

[cgf14793-bib-0003] [ABWB19] Abrevaya V. F. , Boukhayma A. , Wuhrer S. , Boyer E. : A Decoupled 3D Facial Shape Model by Adversarial Training. In 2019 IEEE/CVF International Conference on Computer Vision (ICCV) . IEEE, Seoul, Korea (South) (Oct 2019), pp. 9418–9427.

[cgf14793-bib-0004] [ACB17] Arjovsky M. , Chintala S. , Bottou L. : Wasserstein generative adversarial networks. In Proceedings of the 34th International Conference on Machine Learning . Precup D. , Teh Y. W. , (Eds.), vol. 70 of *Proceedings of Machine Learning Research*, PMLR, Sydney, Australia (Aug 2017), pp. 214–223.

[cgf14793-bib-0005] [ADMG18] Achlioptas P. , Diamanti O. , Mitliagkas I. , Guibas L. : Learning representations and generative models for 3d point clouds. In Proceedings of the 35th International Conference on Machine Learning . Dy J. , Krause A. , (Eds.), vol. 80 of *Proceedings of Machine Learning Research*, PMLR, Stockholm, Sweden (July 2018), pp. 40–49.

[cgf14793-bib-0006] [AW20] Alharbi Y. , Wonka P. : Disentangled image generation through structured noise injection. In Proceedings of the IEEE/CVF Conference on Computer Vision and Pattern Recognition . IEEE, Virtual (2020), pp. 5134–5142.

[cgf14793-bib-0007] [BBP*19] Bouritsas G. , Bokhnyak S. , Ploumpis S. , Bronstein M. , Zafeiriou S. : Neural 3d morphable models: Spiral convolutional networks for 3d shape representation learning and generation. In Proceedings of the IEEE/CVF International Conference on Computer Vision . IEEE, Seoul, Korea (South) (2019), pp. 7213–7222.

[cgf14793-bib-0008] [BCV13] Bengio Y. , Courville A. , Vincent P. : Representation learning: A review and new perspectives. IEEE Transactions on Pattern Analysis and Machine Intelligence 35, 8 (2013), 1798–1828. 23787338 10.1109/TPAMI.2013.50

[cgf14793-bib-0009] [BRZ*16] Booth J. , Roussos A. , Zafeiriou S. , Ponniah A. , Dunaway D. : A 3d morphable model learnt from 10,000 faces. In Proceedings of the IEEE Conference on Computer Vision and Pattern Recognition . IEEE, Las Vegas, Nevada (2016), pp. 5543–5552.

[cgf14793-bib-0010] [BV99] Blanz V. , Vetter T. : A morphable model for the synthesis of 3d faces. In Proceedings of the 26th Annual Conference on Computer Graphics and Interactive Techniques . SIGGRAPH '99, ACM Press/Addison‐Wesley Publishing Co., Los Angeles, California, USA (1999), pp. 187–194.

[cgf14793-bib-0011] [BZSL13] Bruna J. , Zaremba W. , Szlam A. , LeCun Y. : Spectral networks and locally connected networks on graphs. arXiv preprint arXiv:1312.6203 (2013).

[cgf14793-bib-0012] [CBGB20] Chandran P. , Bradley D. , Gross M. , Beeler T. : Semantic deep face models. In 2020 International Conference on 3D Vision (3DV) . IEEE, Fukuoka, Japan (2020), pp. 345–354.

[cgf14793-bib-0013] [CBZ*19] Cheng S. , Bronstein M. , Zhou Y. , Kotsia I. , Pantic M. , Zafeiriou S. : Meshgan: Non‐linear 3d morphable models of faces. arXiv preprint arXiv:1903.10384 (2019).

[cgf14793-bib-0014] [Cha84] Chavel I. : Eigenvalues in Riemannian geometry. Academic Press, Orlando, Florida (1984).

[cgf14793-bib-0015] [CNH*20] Cosmo L. , Norelli A. , Halimi O. , Kimmel R. , Rodola E. : Limp: Learning latent shape representations with metric preservation priors. In European Conference on Computer Vision – ECCV 2020 . Springer, Springer International Publishing (Online, 2020), pp. 19–35.

[cgf14793-bib-0016] [CRW*18] Claes P. , Roosenboom J. , White J. D. , Swigut T. , Sero D. , Li J. , Lee M. K. , Zaidi A. , Mattern B. C. , Liebowitz C. , et al.: Genome‐wide mapping of global‐to‐local genetic effects on human facial shape. Nature genetics 50, 3 (2018), 414–423. 29459680 10.1038/s41588-018-0057-4 PMC5937280

[cgf14793-bib-0017] [CTS*21] Chen H. , Tang H. , Shi H. , Peng W. , Sebe N. , Zhao G. : Intrinsic‐extrinsic preserved gans for unsupervised 3d pose transfer. In Proceedings of the IEEE/CVF International Conference on Computer Vision . IEEE, Virtual (2021), pp. 8630–8639.

[cgf14793-bib-0018] [DBV16] Defferrard M. , Bresson X. , Vandergheynst P. : Convolutional neural networks on graphs with fast localized spectral filtering. In Proceedings of the 30th International Conference on Neural Information Processing Systems . NIPS'16, Curran Associates Inc., Red Hook, NY, USA (2016), p. 3844–3852.

[cgf14793-bib-0019] [DPSD20] Dai H. , Pears N. , Smith W. , Duncan C. : Statistical modeling of craniofacial shape and texture. International Journal of Computer Vision 128, 2 (2020), 547–571.

[cgf14793-bib-0020] [DS19] Dai H. , Shao L. : Pointae: Point auto‐encoder for 3d statistical shape and texture modelling. In Proceedings of the IEEE/CVF International Conference on Computer Vision . IEEE, Seoul, Korea (South) (2019), pp. 5410–5419.

[cgf14793-bib-0021] [DXX*20] Ding Z. , Xu Y. , Xu W. , Parmar G. , Yang Y. , Welling M. , Tu Z. : Guided variational autoencoder for disentanglement learning. In Proceedings of the IEEE/CVF Conference on Computer Vision and Pattern Recognition . IEEE, Virtual (2020), pp. 7920–7929.

[cgf14793-bib-0022] [EST*20] Egger B. , Smith W. A. , Tewari A. , Wuhrer S. , Zollhoefer M. , Beeler T. , Bernard F. , Bolkart T. , Kortylewski A. , Romdhani S. , et al.: 3d morphable face models—past, present, and future. ACM Transactions on Graphics (TOG) 39, 5 (2020), 1–38.

[cgf14793-bib-0023] [EWJ*19] Esmaeili B. , Wu H. , Jain S. , Bozkurt A. , Siddharth N. , Paige B. , Brooks D. H. , Dy J. , Meent J.‐W. : Structured disentangled representations. In The 22nd International Conference on Artificial Intelligence and Statistics . PMLR, PMLR, Naha, Okinawa, Japan (2019), pp. 2525–2534.

[cgf14793-bib-0024] [FKD*20] Foti S. , Koo B. , Dowrick T. , Ramalhinho J. a. , Allam M. , Davidson B. , Stoyanov D. , Clarkson M. J. : Intraoperative liver surface completion with graph convolutional vae. In Uncertainty for Safe Utilization of Machine Learning in Medical Imaging, and Graphs in Biomedical Image Analysis. Springer‐Verlag, Berlin, Heidelberg (2020), pp. 198–207.

[cgf14793-bib-0025] [FKSC22] Foti S. , Koo B. , Stoyanov D. , Clarkson M. J. : 3D shape variational autoencoder latent disentanglement via mini‐batch feature swapping for bodies and faces. In Proceedings of the IEEE/CVF Conference on Computer Vision and Pattern Recognition . IEEE, New Orleans, Louisiana, USA (2022), pp. 18730–18739.

[cgf14793-bib-0026] [GCBZ19] Gong S. , Chen L. , Bronstein M. , Zafeiriou S. : Spiralnet++: A fast and highly efficient mesh convolution operator. In Proceedings of the IEEE/CVF International Conference on Computer Vision Workshops . IEEE, Seoul, Korea (South) (2019).

[cgf14793-bib-0027] [GFZ*20] Gruber A. , Fratarcangeli M. , Zoss G. , Cattaneo R. , Beeler T. , Gross M. , Bradley D. : Interactive sculpting of digital faces using an anatomical modeling paradigm. Computer Graphics Forum 39, 5 (2020), 93–102.

[cgf14793-bib-0028] [GLP*20] Gecer B. , Lattas A. , Ploumpis S. , Deng J. , Papaioannou A. , Moschoglou S. , Zafeiriou S. : Synthesizing coupled 3d face modalities by trunk‐branch generative adversarial networks. In European Conference on Computer Vision . Springer, IEEE, Virtual (2020), pp. 415–433.

[cgf14793-bib-0029] [GYP14] Gao Z. , Yu Z. , Pang X. : A compact shape descriptor for triangular surface meshes. Computer‐Aided Design 53, (2014), 62–69. 24910467 10.1016/j.cad.2014.03.008 PMC4041874

[cgf14793-bib-0030] [HHLP20] Härkönen E. , Hertzmann A. , Lehtinen J. , Paris S. : Ganspace: Discovering interpretable gan controls. Advances in Neural Information Processing Systems 33, (2020), 9841–9850.

[cgf14793-bib-0031] [HHS*21] Huang Q. , Huang X. , Sun B. , Zhang Z. , Jiang J. , Bajaj C. : Arapreg: An as‐rigid‐as possible regularization loss for learning deformable shape generators. In Proceedings of the IEEE/CVF International Conference on Computer Vision . IEEE, Virtual (2021), pp. 5815–5825.

[cgf14793-bib-0032] [HMP*17] Higgins I. , Matthey L. , Pal A. , Burgess C. , Glorot X. , Botvinick M. , Mohamed S. , Lerchner A. : beta‐VAE: Learning basic visual concepts with a constrained variational framework. In International Conference on Learning Representations . Toulon, France (2017).

[cgf14793-bib-0033] [HMWL22] Huang X. , Mallya A. , Wang T.‐C. , Liu M.‐Y. : Multimodal conditional image synthesis with product‐of‐experts gans. In European Conference on Computer Vision . Springer International, Tel Aviv, Israel (2022).

[cgf14793-bib-0034] [HZK*19] He Z. , Zuo W. , Kan M. , Shan S. , Chen X. : Attgan: Facial attribute editing by only changing what you want. IEEE Transactions on Image Processing 28, 11 (2019), 5464–5478. 31107649 10.1109/TIP.2019.2916751

[cgf14793-bib-0035] [JWCZ19] Jiang Z.‐H. , Wu Q. , Chen K. , Zhang J. : Disentangled representation learning for 3d face shape. In Proceedings of the IEEE/CVF Conference on Computer Vision and Pattern Recognition . IEEE, Long Beach, California, USA (2019), pp. 11957–11966.

[cgf14793-bib-0036] [KAL*21] Karras T. , Aittala M. , Laine S. , Härkönen E. , Hellsten J. , Lehtinen J. , Aila T. : Alias‐free generative adversarial networks. In Advances in Neural Information Processing Systems . Curran Associates, Inc., (2021), vol. 34, pp. 852–863.

[cgf14793-bib-0037] [KM18] Kim H. , Mnih A. : Disentangling by factorising. In International Conference on Machine Learning . PMLR, Stockholm, Sweden (2018), pp. 2649–2658.

[cgf14793-bib-0038] [KSB18] Kumar A. , Sattigeri P. , Balakrishnan A. : Variational inference of disentangled latent concepts from unlabeled observations. In International Conference on Learning Representations . Vancouver, Canada (2018).

[cgf14793-bib-0039] [KW14] Kingma D. P. , Welling M. : Auto‐encoding variational bayes. In International Conference on Learning Representations . Banff, Canada (2014).

[cgf14793-bib-0040] [KWKT15] Kulkarni T. D. , Whitney W. F. , Kohli P. , Tenenbaum J. : Deep convolutional inverse graphics network. In Advances in Neural Information Processing Systems . Cortes C. , Lawrence N. , Lee D. , Sugiyama M. , Garnett R. , (Eds.), Curran Associates, Inc., Montreal, Canada (2015), vol. 28.

[cgf14793-bib-0041] [LAR*14] Lewis J. P. , Anjyo K. , Rhee T. , Zhang M. , Pighin F. H. , Deng Z. : Practice and theory of blendshape facial models. Eurographics (State of the Art Reports) 1, 8 (2014), 2.

[cgf14793-bib-0042] [LBB*17] Li T. , Bolkart T. , Black M. J. , Li H. , Romero J. : Learning a model of facial shape and expression from 4d scans. ACM Transections on Graphics 36, 6 (2017), 194–1.

[cgf14793-bib-0043] [LBBM18] Litany O. , Bronstein A. , Bronstein M. , Makadia A. : Deformable shape completion with graph convolutional autoencoders. In Proceedings of the IEEE Conference on Computer Vision and Pattern Recognition . IEEE, Salt Lake City, Utah, USA (2018), pp. 1886–1895.

[cgf14793-bib-0044] [LBZ*20] Li R. , Bladin K. , Zhao Y. , Chinara C. , Ingraham O. , Xiang P. , Ren X. , Prasad P. , Kishore B. , Xing J. , et al.: Learning formation of physically‐based face attributes. In Proceedings of the IEEE/CVF Conference on Computer Vision and Pattern Recognition . CVPR, Virtual (2020), pp. 3410–3419.

[cgf14793-bib-0045] [LKL*21] Ling H. , Kreis K. , Li D. , Kim S. W. , Torralba A. , Fidler S. : Editgan: High‐precision semantic image editing. In Advances in Neural Information Processing Systems . Curran Associates, Inc., Virtual (2021), vol. 34, pp. 16331–16345.

[cgf14793-bib-0046] [LLHF21] Li R. , Li X. , Hui K.‐H. , Fu C.‐W. : Sp‐gan: Sphere‐guided 3d shape generation and manipulation. ACM Transactions on Graphics (TOG) 40, 4 (2021), 1–12.

[cgf14793-bib-0047] [LLW22] Li S. , Liu M. , Walder C. : Editvae: Unsupervised parts‐aware controllable 3d point cloud shape generation. Proceedings of the AAAI Conference on Artificial Intelligence 36, 2 (June 2022), 1386–1394.

[cgf14793-bib-0048] [LLWL20] Lee C.‐H. , Liu Z. , Wu L. , Luo P. : Maskgan: Towards diverse and interactive facial image manipulation. In Proceedings of the IEEE/CVF Conference on Computer Vision and Pattern Recognition . IEEE, Virtual (2020), pp. 5549–5558.

[cgf14793-bib-0049] [LMR*15] Loper M. , Mahmood N. , Romero J. , Pons‐Moll G. , Black M. J. : Smpl: A skinned multi‐person linear model. ACM Transactions on Graphics (TOG) 34, 6 (2015), 1–16.

[cgf14793-bib-0050] [LYF*21] Lombardi S. , Yang B. , Fan T. , Bao H. , Zhang G. , Pollefeys M. , Cui Z. : Latenthuman: Shape‐and‐pose disentangled latent representation for human bodies. In 2021 International Conference on 3D Vision (3DV) . IEEE, Virtual (2021), pp. 278–288.

[cgf14793-bib-0051] [Lyo14] Lyon A. : Why are normal distributions normal? The British Journal for the Philosophy of Science 65, 3 (2014), 621–649.

[cgf14793-bib-0052] [MLX*17] Mao X. , Li Q. , Xie H. , Lau R. Y. , Wang Z. , Paul Smolley S. : Least squares generative adversarial networks. In Proceedings of the IEEE International Conference on Computer Vision . IEEE, Venice, Italy (2017), pp. 2794–2802.

[cgf14793-bib-0053] [MPN*20] Moschoglou S. , Ploumpis S. , Nicolaou M. A. , Papaioannou A. , Zafeiriou S. : 3dfacegan: Adversarial nets for 3d face representation, generation, and translation. International Journal of Computer Vision 128, (2020), 2534–2551.

[cgf14793-bib-0054] [MRC*21] Marin R. , Rampini A. , Castellani U. , Rodolà E. , Ovsjanikov M. , Melzi S. : Spectral shape recovery and analysis via data‐driven connections. International Journal of Computer Vision 129, (2021), 2745–2760. 34720402 10.1007/s11263-021-01492-6 PMC8550494

[cgf14793-bib-0055] [NW17] Nash C. , Williams C. K. I. : The shape variational autoencoder: A deep generative model of part‐segmented 3d objects. Computer Graphics Forum 36, 5 (2017), 1–12.

[cgf14793-bib-0056] [OBB20] Osman A. A. A. , Bolkart T. , Black M. J. : STAR: A sparse trained articulated human body regressor. In European Conference on Computer Vision (ECCV) . Springer International Publishing, Virtual (2020), pp. 598–613.

[cgf14793-bib-0057] [OBD*21] Olivier N. , Baert K. , Danieau F. , Multon F. , Avril Q. : Facetunegan: Face autoencoder for convolutional expression transfer using neural generative adversarial networks. Computer & Graphics, 110 (2023), 69–85.

[cgf14793-bib-0058] [OFD*22] Otberdout N. , Ferrari C. , Daoudi M. , Berretti S. , Del Bimbo A. : Sparse to dense dynamic 3d facial expression generation. In Proceedings of the IEEE/CVF Conference on Computer Vision and Pattern Recognition . IEEE, New Orleans, Louisiana, USA (2022), pp. 20385–20394.

[cgf14793-bib-0059] [OvdLP*22] O'Sullivan E. , van de Lande L. S. , Papaioannou A. , Breakey R. W. , Jeelani N. O. , Ponniah A. , Duncan C. , Schievano S. , Khonsari R. H. , Zafeiriou S. , et al.: Convolutional mesh autoencoders for the 3‐dimensional identification of fgfr‐related craniosynostosis. Scientific reports 12, 1 (2022), 1–8. 35140239 10.1038/s41598-021-02411-y PMC8828904

[cgf14793-bib-0060] [PMRMB15] Pons‐Moll G. , Romero J. , Mahmood N. , Black M. J. : Dyna: A model of dynamic human shape in motion. ACM Transactions on Graphics (TOG) 34, 4 (2015), 1–14.

[cgf14793-bib-0061] [PVS*21] Ploumpis S. , Ververas E. , Sullivan E. O. , Moschoglou S. , Wang H. , Pears N. , Smith W. A. P. , Gecer B. , Zafeiriou S. : Towards a complete 3d morphable model of the human head. IEEE Transactions on Pattern Analysis and Machine Intelligence 43, 11 (2021), 4142–4160. 32356737 10.1109/TPAMI.2020.2991150

[cgf14793-bib-0062] [PWP*19] Ploumpis S. , Wang H. , Pears N. , Smith W. A. , Zafeiriou S. : Combining 3d morphable models: A large scale face‐and‐head model. In Proceedings of the IEEE Conference on Computer Vision and Pattern Recognition . IEEE, Long Beach, California, USA (2019), pp. 10934–10943.

[cgf14793-bib-0063] [RBSB18] Ranjan A. , Bolkart T. , Sanyal S. , Black M. J. : Generating 3d faces using convolutional mesh autoencoders. In Proceedings of the European Conference on Computer Vision (ECCV) . Springer International Publishing, Munich, Germany (2018), pp. 704–720.

[cgf14793-bib-0064] [RDC*21] Roberts D. , Danielyan A. , Chu H. , Golparvar‐Fard M. , Forsyth D. : Lsd‐structurenet: Modeling levels of structural detail in 3d part hierarchies. In Proceedings of the IEEE/CVF International Conference on Computer Vision . IEEE, Virtual (2021), pp. 5836–5845.

[cgf14793-bib-0065] [RKH*21] Radford A. , Kim J. W. , Hallacy C. , Ramesh A. , Goh G. , Agarwal S. , Sastry G. , Askell A. , Mishkin P. , Clark J. , Krueger G. , Sutskever I. : Learning transferable visual models from natural language supervision. In Proceedings of the 38th International Conference on Machine Learning . Meila M. , Zhang T. , (Eds.), vol. 139 of *Proceedings of Machine Learning Research*, PMLR, Virtual (2021), pp. 8748–8763.

[cgf14793-bib-0066] [RL21] Rhodes T. , Lee D. : Local disentanglement in variational auto‐encoders using jacobian l_1 regularization. In Advances in Neural Information Processing Systems . Curran Associates, Inc., Virtual (2021), vol. 34.

[cgf14793-bib-0067] [RWP06] Reuter M. , Wolter F.‐E. , Peinecke N. : Laplace–beltrami spectra as ‘shape‐dna’ of surfaces and solids. Computer‐Aided Design 38, 4 (2006), 342–366.

[cgf14793-bib-0068] [SBKM21] Shoshan A. , Bhonker N. , Kviatkovsky I. , Medioni G. : Gan‐control: Explicitly controllable gans. In Proceedings of the IEEE/CVF International Conference on Computer Vision . IEEE, Virtual (2021), pp. 14083–14093.

[cgf14793-bib-0069] [SNF*13] Shuman D. I. , Narang S. K. , Frossard P. , Ortega A. , Vandergheynst P. : The emerging field of signal processing on graphs: Extending high‐dimensional data analysis to networks and other irregular domains. IEEE Signal Processing Magazine 30, 3 (2013), 83–98.

[cgf14793-bib-0070] [SYTZ22] Shen Y. , Yang C. , Tang X. , Zhou B. : Interfacegan: Interpreting the disentangled face representation learned by gans. IEEE Transactions on Pattern Analysis and Machine Intelligence 44, 4 (2022), 2004–2018. 33108282 10.1109/TPAMI.2020.3034267

[cgf14793-bib-0071] [TDlTM11] Tena J. R. , De la Torre F. , Matthews I. : Interactive region‐based linear 3d face models. ACM Transactions on Graphics 30, 4 (7 2011).

[cgf14793-bib-0072] [TSL21] Tatro N. J. , Schonsheck S. C. , Lai R. : Unsupervised geometric disentanglement via CFAN‐VAE. ICLR Workshop on Geometrical and Topological Representation Learning. Virtual (2021).

[cgf14793-bib-0073] [TZY*22] Tan Q. , Zhang L.‐X. , Yang J. , Lai Y.‐K. , Gao L. : Variational autoencoders for localized mesh deformation component analysis. IEEE Transactions on Pattern Analysis and Machine Intelligence 44, 10 (2022), 6297–6310. 34061742 10.1109/TPAMI.2021.3085887

[cgf14793-bib-0074] [VB20] Voynov A. , Babenko A. : Unsupervised discovery of interpretable directions in the gan latent space. In International Conference on Machine Learning . PMLR, PMLR, Virtual, (2020), pp. 9786–9796.

[cgf14793-bib-0075] [VRM*17] Varol G. , Romero J. , Martin X. , Mahmood N. , Black M. J. , Laptev I. , Schmid C. : Learning from synthetic humans. In Proceedings of the IEEE Conference on Computer Vision and Pattern Recognition . IEEE, Honolulu, Hawaii, USA (2017), pp. 109–117.

[cgf14793-bib-0076] [WDH*19] Wang W. , Dang Z. , Hu Y. , Fua P. , Salzmann M. : Backpropagation‐friendly eigendecomposition. In Advances in Neural Information Processing Systems . Virtual, (2019), vol. 32.

[cgf14793-bib-0077] [WYH*21] Wang T. , Yue Z. , Huang J. , Sun Q. , Zhang H. : Self‐supervised learning disentangled group representation as feature. In Advances in Neural Information Processing Systems . Curran Associates, Inc., Virtual (2021), vol. 34.

[cgf14793-bib-0078] [YFST18] Yang Y. , Feng C. , Shen Y. , Tian D. : Foldingnet: Point cloud auto‐encoder via deep grid deformation. In Proceedings of the IEEE Conference on Computer Vision and Pattern Recognition . IEEE, Salt Lake City, Utah, USA (2018), pp. 206–215.

[cgf14793-bib-0079] [YHH*19] Yang G. , Huang X. , Hao Z. , Liu M.‐Y. , Belongie S. , Hariharan B. : Pointflow: 3d point cloud generation with continuous normalizing flows. In Proceedings of the IEEE/CVF International Conference on Computer Vision . IEEE, Seoul, Korea (South) (2019), pp. 4541–4550.

[cgf14793-bib-0080] [YLY*20] Yuan Y.‐J. , Lai Y.‐K. , Yang J. , Duan Q. , Fu H. , Gao L. : Mesh variational autoencoders with edge contraction pooling. In Proceedings of the IEEE/CVF Conference on Computer Vision and Pattern Recognition Workshops . IEEE, Virtual (2020), pp. 274–275.

[cgf14793-bib-0081] [YML*20] Yang J. , Mo K. , Lai Y.‐K. , Guibas L. J. , Gao L. : DSG‐Net: learning disentangled structure and geometry for 3D shape generation. ACM Transactions on Graphics 42, 1 (2022), 1–17.

[cgf14793-bib-0082] [ZBPM20] Zhou K. , Bhatnagar B. L. , Pons‐Moll G. : Unsupervised shape and pose disentanglement for 3d meshes. In European Conference on Computer Vision . Springer, Springer International Publishing, Virtual (2020), pp. 341–357.

[cgf14793-bib-0083] [ZKJB17] Zuffi S. , Kanazawa A. , Jacobs D. W. , Black M. J. : 3d menagerie: Modeling the 3d shape and pose of animals. In Proceedings of the IEEE Conference on Computer Vision and Pattern Recognition . IEEE, Honolulu, Hawaii, USA (2017), pp. 6365–6373.

[cgf14793-bib-0084] [ZVKD10] Zhang H. , Van Kaick O. , Dyer R. : Spectral mesh processing. Computer Graphics Forum 29, 6 (2010), 1865–1894.

[cgf14793-bib-0085] [ZWL*20] Zhou Y. , Wu C. , Li Z. , Cao C. , Ye Y. , Saragih J. , Li H. , Sheikh Y. : Fully convolutional mesh autoencoder using efficient spatially varying kernels. Advances in Neural Information Processing Systems 33, (2020), 9251–9262.

[cgf14793-bib-0086] [ZXT20] Zhu X. , Xu C. , Tao D. : Learning disentangled representations with latent variation predictability. In European Conference on Computer Vision . Springer International Publishing, Virtual (2020), pp. 684–700.

[cgf14793-bib-0087] [ZYHC22] Zheng M. , Yang H. , Huang D. , Chen L. : Imface: A nonlinear 3d morphable face model with implicit neural representations. In Proceedings of the IEEE/CVF Conference on Computer Vision and Pattern Recognition . IEEE, New Orleans, Louisiana, USA (2022), pp. 20343–20352.

[cgf14793-bib-0088] [ZYL*20] Zhang Z. , Yu C. , Li H. , Sun J. , Liu F. : Learning distribution independent latent representation for 3d face disentanglement. In 2020 International Conference on 3D Vision (3DV) . IEEE, Virtual (2020), pp. 848–857.

