# Supplementary material for: 3D Generative Model Latent Disentanglement via Local Eigenprojection
Source: Comput Graph Forum. 2023 Apr 4;42(6):e14793. doi: 10.1111/cgf.14793 (PMC10617979; doi:10.1111/cgf.14793)
Supplement: Supplementary file 2 — Supporting Information [file CGF-42-e14793-s002.pdf]

Volume 0 (1981), Number 0 pp. 1–4 COMPUTER GRAPHICS *forum*

Supplementary Materials of: 3D Generative Model Latent Disentanglement via Local Eigenprojection

Simone Foti[1], Bongjin Koo[1,2], Danail Stoyanov[1], and Matthew J. Clarkson[1]

[1]University College London, London, UK
[2]University of California, Santa Barbara, Santa Barbara, USA

1. PCA-Based Baseline

LED models and SD-VAE are characterised by a single architecture that, thanks to a disentangled latent representation, can control the generation of local shape attributes while still considering the whole output shape. To demonstrate the need for these models we compare our method also against a bundle of attribute-specific PCA models. As it can be observed in Fig. 8, the main issue of naive per-part methods, such as the bundle of PCA models, is that shape attributes are independently generated. Even though this makes the different attributes fully disentangled between each others, significant surface discontinuities appear during the generation procedure. On the contrary, LED models are capable of ensuring the continuity of the output surfaces while providing control over the generation of each attribute.

2. Mesh Operators

Traditional neural network operators are not well suited for non-Euclidean data such as meshes. In recent years, many operators capable of operating on meshes were proposed. We decide to build all models with the intuitive spiral++ convolutions and with quadric-sampling-based pooling operators as in [FKSC22; GCBZ19]. However, other mesh operators could be used. Spiral++ convolutions, which are specifically designed to efficiently operate on datasets of meshes sharing the same topology [GCBZ19], are built aggregating vertices along spiral sequences and processing them with a multilayer perceptron. Spirals are defined for each vertex of the mesh by selecting an arbitrary neighbour as well as the other vertices along a clockwise spiral. All spirals are precomputed and have a fixed length. The receptive field of these convolutions can be increased by dilating the spirals, thus skipping a predefined amount of vertices after each selected vertex. Since spirals are precomputed, only the multilayer preceptron's weights are learned during training. Also the pooling operators are precomputed. In fact, a quadric sampling procedure that iteratively contracts the vertex pair with the smallest quadric error is applied to the mean shape of the training data $\mathbf{M}$. During this procedure both a pooling and an un-pooling sparse matrix are defined. The former has values of 1 in correspondence of vertices that need to be preserved and 0 elsewhere. The latter still has values of 1 for vertices that remain unchanged while un-pooling, but it also stores the barycentric weights corresponding to the barycentric coordinates of contracted vertices in order to restore them. These precomputed sparse matrices are matrix multiplied with the vertex features computed by the different network's layers to achieve pooling and un-pooling.

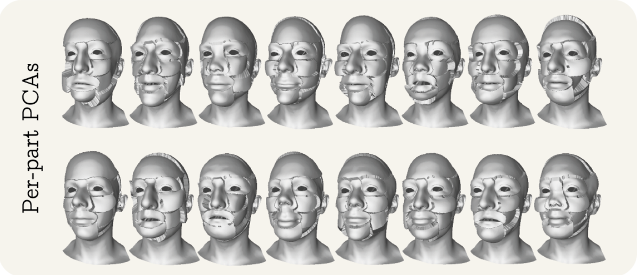

Figure 8: *Random samples generated by a bundle of PCA models trained each on a different shape attribute.*

3. Architectures

VAEs operating on meshes from UHM. The architecture of encoder and generator are defined as:

$$E = e(\text{Conv}(32)) \xrightarrow{\downarrow 4} e(\text{Conv}(32)) \xrightarrow{\downarrow 4} e(\text{Conv}(32)) \xrightarrow{\downarrow 4} e(\text{Conv}(64)) \xrightarrow{\downarrow 4} 2 \times \text{Lin}(60)$$

$$G = \text{Lin}(64n) \xrightarrow{\uparrow 4} e(\text{Conv}(64)) \xrightarrow{\uparrow 4} e(\text{Conv}(32)) \xrightarrow{\uparrow 4} e(\text{Conv}(32)) \xrightarrow{\uparrow 4} e(\text{Conv}(32)) \rightarrow \text{Conv}(3),$$

where $\text{Lin}(\cdot)$ and $\text{Conv}(\cdot)$ respectively represent linear layers and spiral convolutions (Sec. 2) with their number of output features. $e(\cdot)$ is the ELU (exponential linear unit) non-linear activation function. Right arrows represent pooling operations (Sec. 2). Their superscript indicates the sampling factor as well as whether it is an

© 2023 The Author(s)
Computer Graphics Forum © 2023 The Eurographics Association and John Wiley & Sons Ltd. Published by John Wiley & Sons Ltd.

up-sampling (↑) or a down-sampling (↓) operator. n is the number of down-sampled vertices after all the sampling operations. In this case, being N the number of input and output vertices, $n = N/4^4$. Note that E terminates with two linear layers that are responsible to predict $\boldsymbol{\mu}$ and $\boldsymbol{\sigma}$, which are used in conjunction with the reparametrization trick to sample a latent vector $\mathbf{z}$. This vector is the input to the generator G.

The architecture described above is used by VAE, LED-VAE, SD-VAE, and DIP-VAE-I.

VAEs operating on meshes from LYHM and CoMA. Since meshes from LYHM and CoMA have fewer vertices than UHM, the pooling layers of the VAE models have smaller sampling factor. Therefore, we have:

$$E = e(\text{Conv}(32)) \xrightarrow{\downarrow 4} e(\text{Conv}(32)) \xrightarrow{\downarrow 2} e(\text{Conv}(32)) \xrightarrow{\downarrow 2} e(\text{Conv}(64)) \xrightarrow{\downarrow 2} 2 \times \text{Lin}(60)$$

$$G = \text{Lin}(64n) \xrightarrow{\uparrow 2} e(\text{Conv}(64)) \xrightarrow{\uparrow 2} e(\text{Conv}(32)) \xrightarrow{\uparrow 2} e(\text{Conv}(32)) \xrightarrow{\uparrow 4} e(\text{Conv}(32)) \rightarrow \text{Conv}(3).$$

VAEs operating on meshes from STAR. Also STAR has fewer vertices than UHM. In this case we use the same architecture used by [FKSC22]. on this dataset:

$$E = e(\text{Conv}(32)) \xrightarrow{\downarrow 4} e(\text{Conv}(32)) \xrightarrow{\downarrow 4} e(\text{Conv}(64)) \xrightarrow{\downarrow 4} 2 \times \text{Lin}(60)$$

$$G = \text{Lin}(64n) \xrightarrow{\uparrow 4} e(\text{Conv}(64)) \xrightarrow{\uparrow 4} e(\text{Conv}(32)) \xrightarrow{\uparrow 4} e(\text{Conv}(32)) \rightarrow \text{Conv}(3).$$

LSGANs operating on meshes from UHM. As mentioned in Sec. 3, the architecture of G remains the same of the VAEs operating on meshes from UHM and the architecture of the discriminator D is similar to the one of E with some minor difference:

$$G = \text{Lin}(64n) \xrightarrow{\uparrow 4} e(\text{Conv}(64)) \xrightarrow{\uparrow 4} e(\text{Conv}(32)) \xrightarrow{\uparrow 4} e(\text{Conv}(32)) \xrightarrow{\uparrow 4} e(\text{Conv}(32)) \rightarrow \text{Conv}(3)$$

$$D = e(\text{Conv}(32)) \xrightarrow{\downarrow 4} e(\text{Conv}(32)) \xrightarrow{\downarrow 4} e(\text{Conv}(32)) \xrightarrow{\downarrow 4} e(\text{Conv}(64)) \xrightarrow{\downarrow 4} \text{Lin}(60).$$

Note that the main difference between E and D is the number of linear layers at the end of the architecture. This architecture is used for both LSGAN and LED-LSGAN.

WGANs operating on meshes from UHM. The architecture of the generator in WGAN is the same as LSGAN's. The architecture of the critic C is the same as D with fewer neurons in the last linear layer, which outputs a single value. In fact, we have:

$$G = \text{Lin}(64n) \xrightarrow{\uparrow 4} e(\text{Conv}(64)) \xrightarrow{\uparrow 4} e(\text{Conv}(32)) \xrightarrow{\uparrow 4} e(\text{Conv}(32)) \xrightarrow{\uparrow 4} e(\text{Conv}(32)) \rightarrow \text{Conv}(3)$$

$$C = e(\text{Conv}(32)) \xrightarrow{\downarrow 4} e(\text{Conv}(32)) \xrightarrow{\downarrow 4} e(\text{Conv}(32)) \xrightarrow{\downarrow 4} e(\text{Conv}(64)) \xrightarrow{\downarrow 4} \text{Lin}(1).$$

This architecture is used for both WGAN and LED-WGAN.

4. Implementation Details

All networks are implemented in PyTorch using the mesh operators described in Sec. 2 with the implementation made available by [FKSC22] †. We segment heads from UHM, LYHM, and CoMA in $F = 12$ head attributes and split the latent representation $\mathbf{z}$ in F subsets of size $\kappa = 5$. Bodies from STAR have $F = 11$ and $\kappa = 3$. For the sake of comparison, all models trained on the meshes obtained from UHM, CoMA, and STAR are trained for 40 epochs. Since LYHM has significantly fewer meshes, models trained on this dataset are trained for 400 epochs, which correspond to approximately the same number of iterations as other models. In addition, the batch size is always set to 16 while spirals length and dilation are set to 9 and 1 respectively. Data are always standardised by subtracting the per-vertex mean of the training set ($\mathbf{M}$) and dividing by the per-vertex standard deviation of the training set ($\boldsymbol{\Sigma}$).

VAEs. All VAE models are trained with the ADAM optimizer using a fixed learning rate of $1e^{-4}$ and a KL divergence weight set to $\beta = 1e^{-4}$. The vanilla VAE, DIP-VAE-I, and SD-VAE have a smoothing loss weight of $\alpha = 1$. As reported in [FKSC22], the latent consistency weight of the SD-VAE model is set to 1 and the contrastive margins to 0.5. In DIP-VAE-I we set $\lambda_d = 100$ and $\lambda_{od} = 10$.

LED-VAEs. During the eigendecomposition of the Kirchoff Laplacians $\mathbf{K}_\omega$ we compute the first $K = 50$ eigenvectors. The α weight controlling the smoothing loss is set to $\alpha = 50$, while the weights of the local eigenprojection losses are set to $\eta_1 = 1$ and $\eta_2 = 0.5$. As previously mentioned, the weight controlling the KL divergence is set to $\beta = 1e^{-4}$. The values reported above are used when LED-VAE is trained on the meshes from UHM. When LED-VAE is trained on LYHM, we have: $K = 45$, $\alpha = 10$, $\beta = 1e^{-4}$, $\eta_1 = 0.5$ and $\eta_2 = 0.25$. On CoMA we have: $K = 45$, $\alpha = 50$, $\beta = 1e^{-4}$, $\eta_1 = 1$ and $\eta_2 = 2$. On STAR we have: $K = 50$, $\alpha = 10$, $\beta = 1e^{-4}$, $\eta_1 = 0.1$ and $\eta_2 = 2$.

LSGAN and LED-LSGAN. The generator G is trained using the ADAM optimizer with a fixed learning rate of $1e^{-4}$. The discriminator D using SGD with a fixed learning rate of $8e^{-4}$. The weight of the Laplacian smoothing term is set to $\alpha = 10$ in LSGAN and $\alpha = 50$ in LED-LSGAN. In LED-LSGAN the local eigenprojection loss weight is set to $\eta = 0.5$ and $K = 50$ eigenvalues are computed during the eigendecompositions of the $\mathbf{K}_\omega$.

WGAN and LED-WGAN. Both generator and critc are trained using the RMSprop optimizer. The learning rate of the optimizer

† The SD-VAE, DIP-VAE, and FactorVAE implementations as well as the evaluation code are publicly available at github.com/simofoti/3DVAE-SwapDisentangled

© 2023 The Author(s)
Computer Graphics Forum © 2023 The Eurographics Association and John Wiley & Sons Ltd.

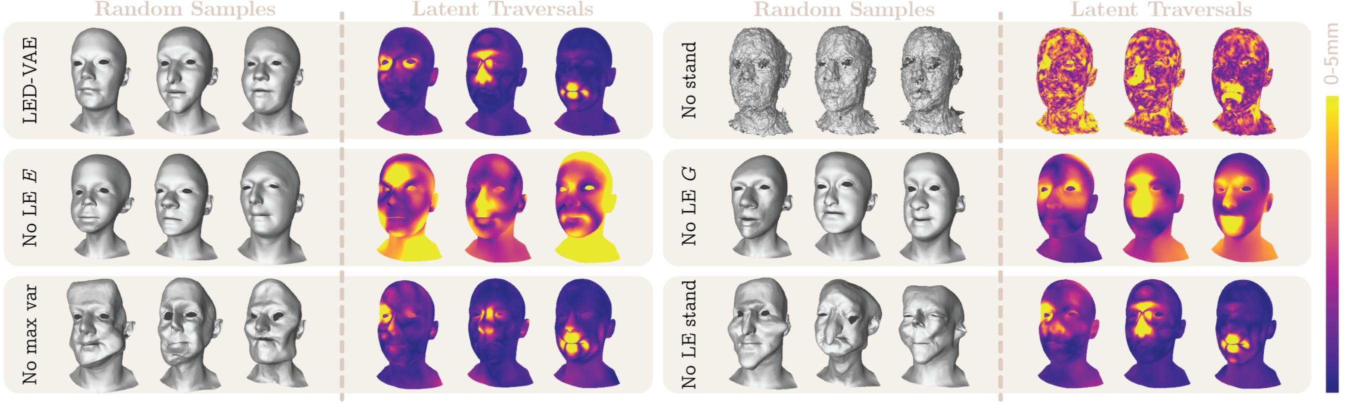

Figure 9: *Ablation study. The LED-VAE is ablated by removing the data standardisation and the computation of the local eigenprojection loss on either the encoder or generator. Albations are performed also selecting the first eigenvectors instead of those associated with the maximum variance and without standardising the local eigenprojections in the loss computation.*

operating on G is set to $1e^{-4}$, while the learning rate of the optimizer operating on C is $5e^{-5}$. The C network weights are clipped to the range $[-c, c]$ with $c = 0.01$. The weight of the Laplacian smoothing term is set to $\alpha = 10$ in WGAN and $\alpha = 50$ in LED-WGAN. In LED-WGAN the local eigenprojection loss weight is set to $\eta = 0.25$ and $K = 50$ eigenvalues are computed.

5. LED-VAE Additional Experiments

As mentioned in Sec. 5, we consider LED-VAE to be the most promising generative model among the proposed LED models because it is simpler to train, requires less hyperparameter tuning, and generates higher quality meshes. Therefore, we conduct experiments to evaluate the importance of the different assumptions made in its construction (Sec. 5.1 and Sec. 5.2), and observe the smoothness of the latent space (Sec. 5.3).

5.1. Ablation Study

The ablation study in Fig. 9 is performed by re-training the proposed LED-VAE without some of its characterising design choices. Models are re-trained with the same architecture (Sec. 3) and implementation details (Sec. 4) of LED-VAE. Only one design choice is altered per ablation experiment. Not standardising the data (No stand in Fig. 9) we observe noisy random samples. Some control over the generation of local attributes appears to be retained, but the presence of noise contributes to shape variations across the entire shape during latent traversals. When the local eigenprojection loss is not computed on the encoder ($\eta_1 = 0$), not only the encoder loses its disentanglement capabilities, but the control over the generation of local shape attributes is significantly reduced (No LE E in Fig. 9). Similar results are obtained when the local eigenprojection loss is not computed on the generator ($\eta_2 = 0$), though the encoder should retain its disentanglement power (No LE G in Fig. 9). If instead of selecting the κ eigenvectors corresponding to the spectral components with the highest variance, we select the first κ eigenvectors as Fourier modes for the local eigenprojection, the generator creates unrealistic shapes (No max var in Fig. 9). Unrealistic shapes are generated also if the local eigenprojections are not standardised and thus $\mathbf{m}^\star_\omega = 0$ and $\mathbf{s}^\star_\omega = 1$ in Eq. 3 (No LE stand in Fig. 9). In addition, note that the vanilla VAE is equivalent to the LED-VAE without local eigenprojection losses and its results are equivalent to those of an ablation experiment where both the local eigenprojection losses are set to zero.

Not only we perform an ablation study removing some charactertising design choice of LED-VAE, but we also experiment with the strength of their weighting coefficients. In Fig. 10, we observe the effects caused by changing the smoothing weight (α). As expected, reducing α reduces also the quality of the randomly generated samples. Interestingly, also the disentanglement performance slightly deteriorates. Increasing α does not have major effects on

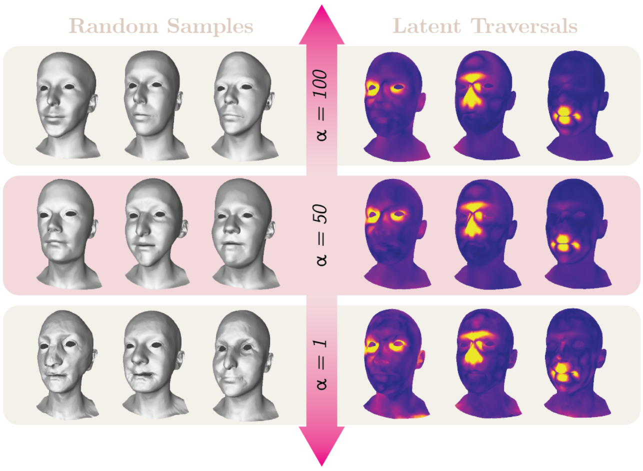

Figure 10: *Effects of smoothness weight (α) on random samples and latent traversals. The row highlighted in pink reports results obtained with the proposed implementation of LED-VAE. Latent traversals are referred always to the same latent variable.*

© 2023 The Author(s)
Computer Graphics Forum © 2023 The Eurographics Association and John Wiley & Sons Ltd.

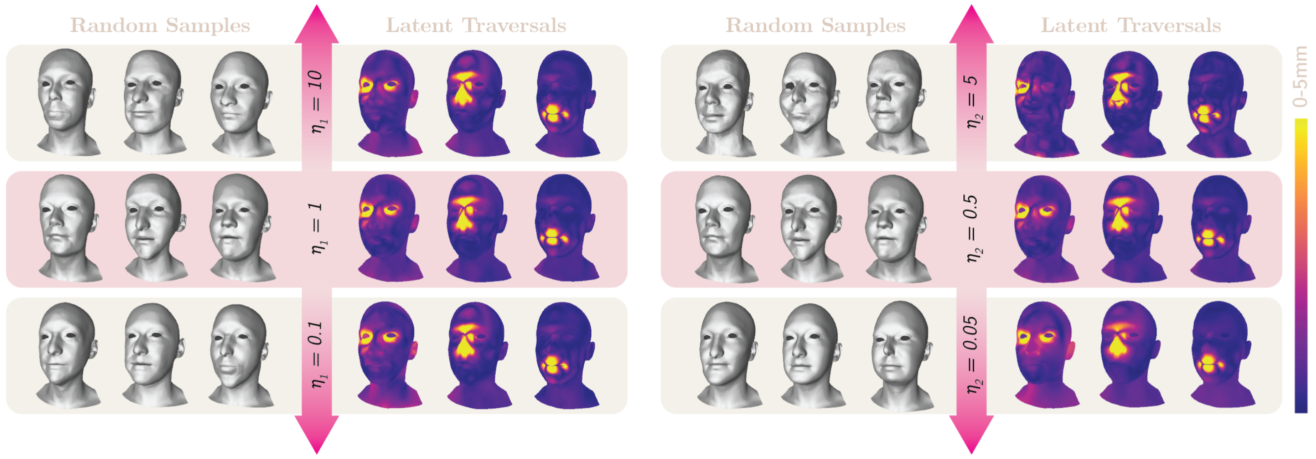

Figure 11: *Effects of local eigenprojection weights on random samples and latent traversals. Rows highlighted in pink report results obtained with the proposed implementation of LED-VAE. The left column shows the effects of changing η_1, which controls the local eigenprojection loss affecting the encoder. The right column the effects of changing η_2, which controls the local eigenprojection loss affecting the generator. Latent traversals are referred always to the same latent variable.*

the generation and disentanglement performance. In Fig. 11, we report the effects of altering the local eigenprojection weighting coefficients. Even though Fig. 9 (No LE E) shows the importance of enforcing the local eigenprojection loss on the encoder, we do not observe significant difference when altering its weight (η_1). Most differences can be appreciated in the first latent traversal, showing how lower weights slightly deteriorate disentanglement. On the contrary, η_2, which modulates the disentanglement on the generator, has more influence on sample quality and disentanglement. In fact, high η_2 values improve disentanglement, but reduce sample quality.

5.2. Different segmentations

The segmentations in Fig. 3 were performed with clinical supervision and were aimed at identifying key anatomical areas of the face and body. Nevertheless, different segmentations are admissible. To observe the disentanglement performance of LED-VAE with different segmentations, we re-trained LED-VAE using a coarser and a finer segmentation. As shown in Fig. 12, LED-VAE successfully disentangles local identity attributes when varying the size and number of local shape attributes.

Note that the local segments are used only by the local eigenprojection losses and are not an input to the network. For this reason, attributes can be connected, overlapping or even not connected. The segmentation used to train our models is connected. Overlapping segments could be used, but big overlaps may be counterproductive as they would increase entanglement between neighbouring regions and may produce unexpected results when eigenprojections of overlapping regions are incompatible.

5.3. Latent Interpolations and Replacements

We perform two latent interpolation experiments and compare results between LED-VAE and SD-VAE, the two variational autoencoders providing control over the generation of local shape attributes. Two randomly selected test shapes $\mathbf{X}_{\text{start}}$ and $\mathbf{X}_{\text{finish}}$ are encoded to compute their respective latent representations. Fig. 13 shows the reconstructed shapes $\mathbf{X}'_{\text{start}}$ and $\mathbf{X}'_{\text{finish}}$ as well as the shapes generated from latent vectors linearly interpolated between the latents of $\mathbf{X}_{\text{start}}$ and $\mathbf{X}_{\text{finish}}$. Fig. 14, Fig. 15, and Fig. 16 depict the effects of changing each $\mathbf{z}_\omega$ of $\mathbf{X}_{\text{start}}$ with the corresponding $\mathbf{z}_\omega$ of $\mathbf{X}_{\text{finish}}$. This is equivalent to progressively replacing attributes of the initial mesh with those of the final mesh. The experiment in Fig. 14 is better represented in the *supplementary video*, where each $\mathbf{z}_\omega$ is interpolated instead of being replaced. These experiments show that the latent space of our LED-VAE is smooth. Even though some self-intersections is visible on the ears of heads generated by LED-VAE, this model appears to be better than SD-VAE at replacing attributes.

6. Random Generation and Latent Disentanglement

We report more randomly generated shapes and latent traversals than those already depicted in Fig. 4 and Fig. 6. In fact, in Fig. 18 we show shapes obtained by all methods trained on heads from UHM and in Fig. 17 shapes generated with LED-VAE trained on LYHM, and CoMA, and STAR. Then we report the effects caused in the generated shapes by traversing all the latent variables. In particular, Fig. 19 shows the shapes generated by traversing all 5 latent variables in each $\mathbf{z}_\omega$ for LED-VAE, SD-VAE, LED-LSGAN, and LED-WGAN. Fig. 20 represents the effects of latent traversals for methods that are not able to enforce disentanglement with respect to local shape attributes, such as: VAE, DIP-VAE-I, LSGAN, and WGAN. Finally, Fig. 21 reports latent traversal results for LED-VAE trained on LYHM, and CoMA, and STAR.

© 2023 The Author(s)
Computer Graphics Forum © 2023 The Eurographics Association and John Wiley & Sons Ltd.

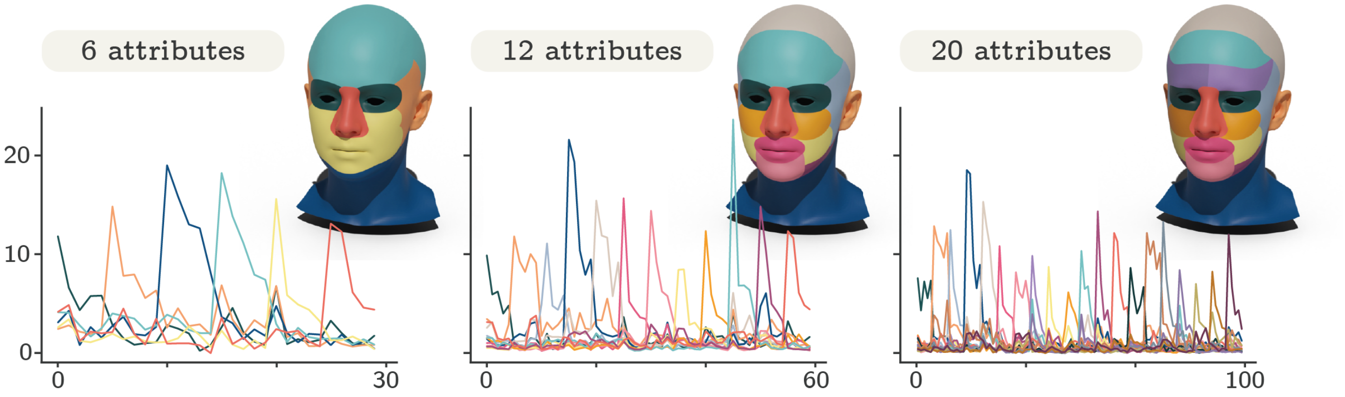

Figure 12: *Effects of traversing each latent variable of LED-VAE trained enforcing latent disentanglement with different attribute segmentations. Note that since* 5 *latent variables are used to represent each attribute, the latent size with* 6 *attributes is equal to* 30*,* 60 *with* 12*, and* 100 *with* 20*. When* 20 *attributes are disentangled, not only we segment the supraorbital area, but we also separate the left from the right attribute. For instance, while with* 12 *attributes left and right eye were grouped together, now they are separate.*

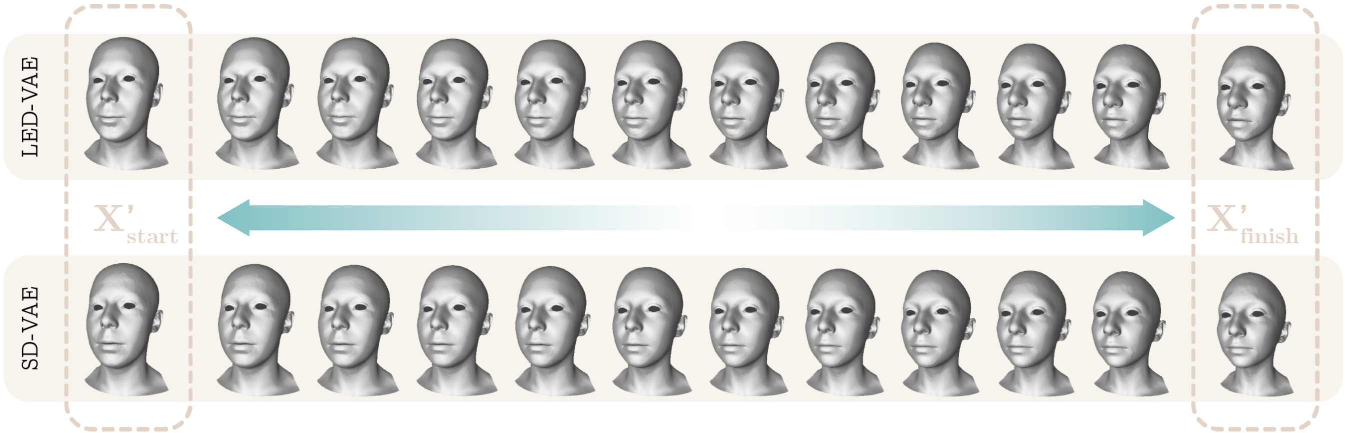

Figure 13: *Latent interpolations with LED-VAE and SD-VAE. Two shapes* ($\mathbf{X}_{start}$ *and* $\mathbf{X}_{finish}$) *are randomly selected from the test set. Their latent representation is computed by feeding the two shapes in the encoder network.* 10 *intermediate latent vectors are obtained by linearly interpolating all the latent variables. Shapes generated from these latent vectors smoothly transition from the reconstructed initial* ($\mathbf{X}'_{start}$) *and final shapes* ($\mathbf{X}'_{finish}$)*.*

© 2023 The Author(s)
Computer Graphics Forum © 2023 The Eurographics Association and John Wiley & Sons Ltd.

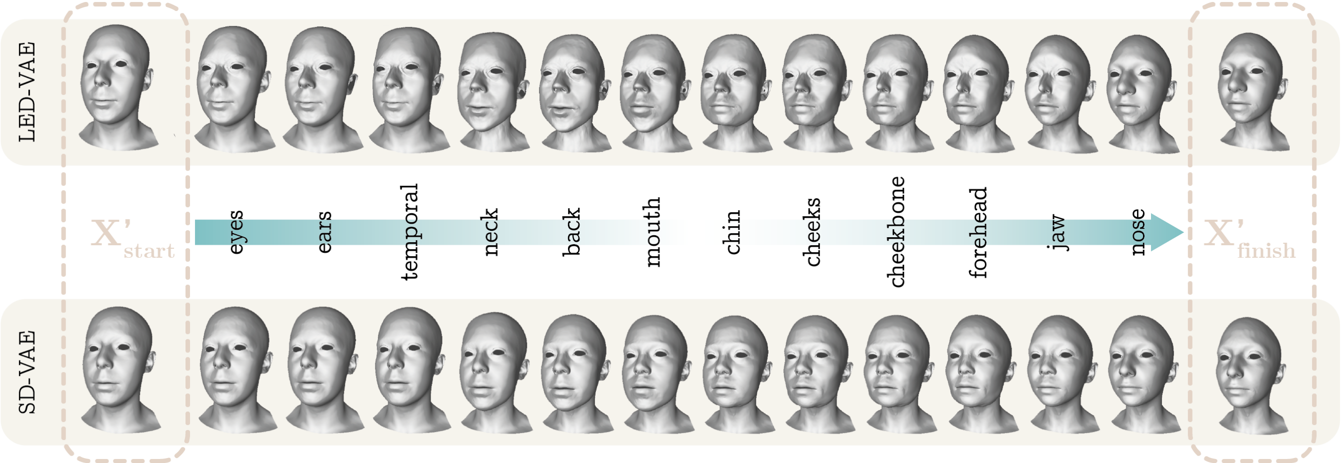

Figure 14: *Per-attribute latent replacements with LED-VAE and SD-VAE. Subsets of the latent variables corresponding to different head attributes ($\mathbf{z}_\omega$) are progressively replaced. While the left-most and right-most heads are the reconstruction of the initial and target shape, the others are obtained with latent replacements. Each shape is generated starting from the one on its left. For example, the second heads from the left are generated with the latent vector of $\mathbf{X}'_{start}$ and replacing the subset of latent variables controlling the eyes of $\mathbf{X}'_{start}$ with the subset controlling the eyes of $\mathbf{X}'_{finish}$. Similarly, the third head has the same latent representation of the second one, but also the subset of latent variables controlling the ears is replaced. The remaining shapes are obtained repeating the same procedure.*

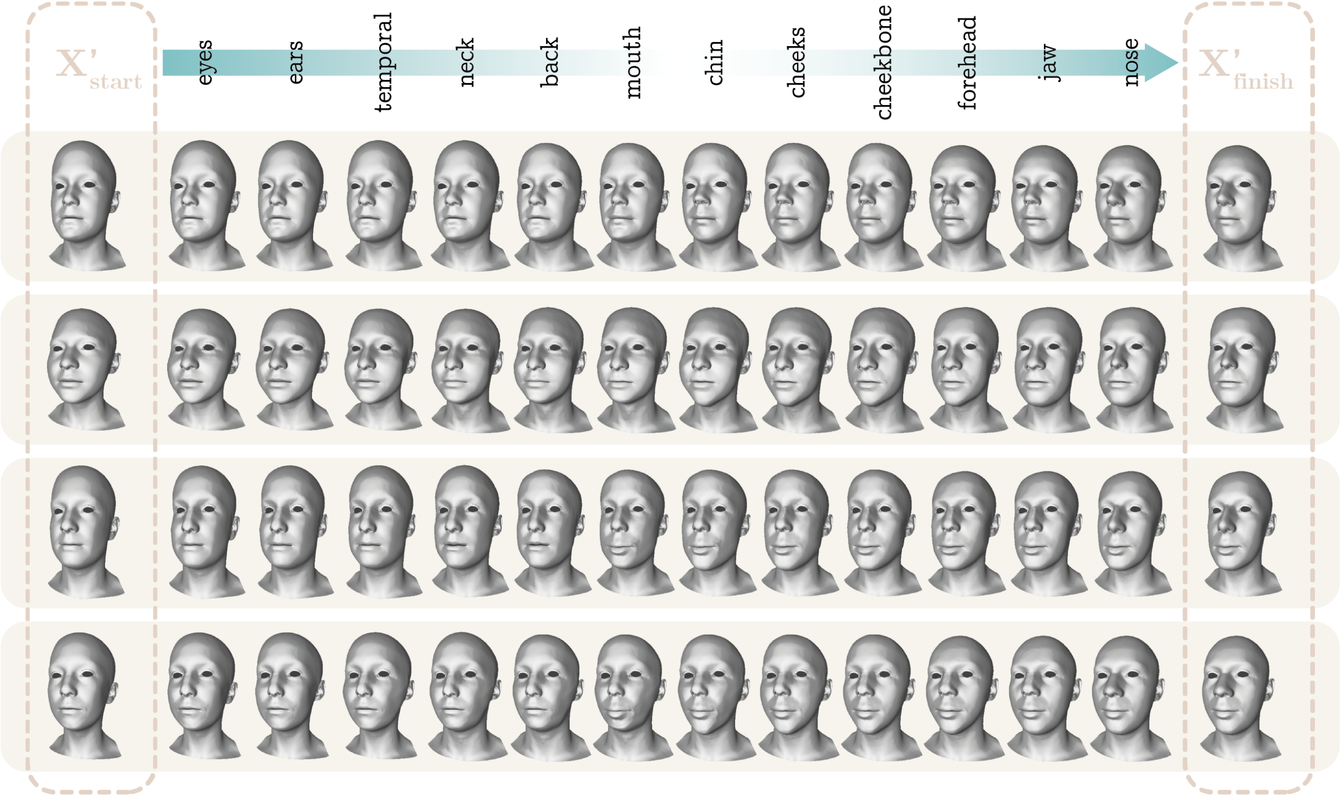

Figure 15: *Additional per-attribute latent replacements with LED-VAE (see Fig. 14).*

© 2023 The Author(s)
Computer Graphics Forum © 2023 The Eurographics Association and John Wiley & Sons Ltd.

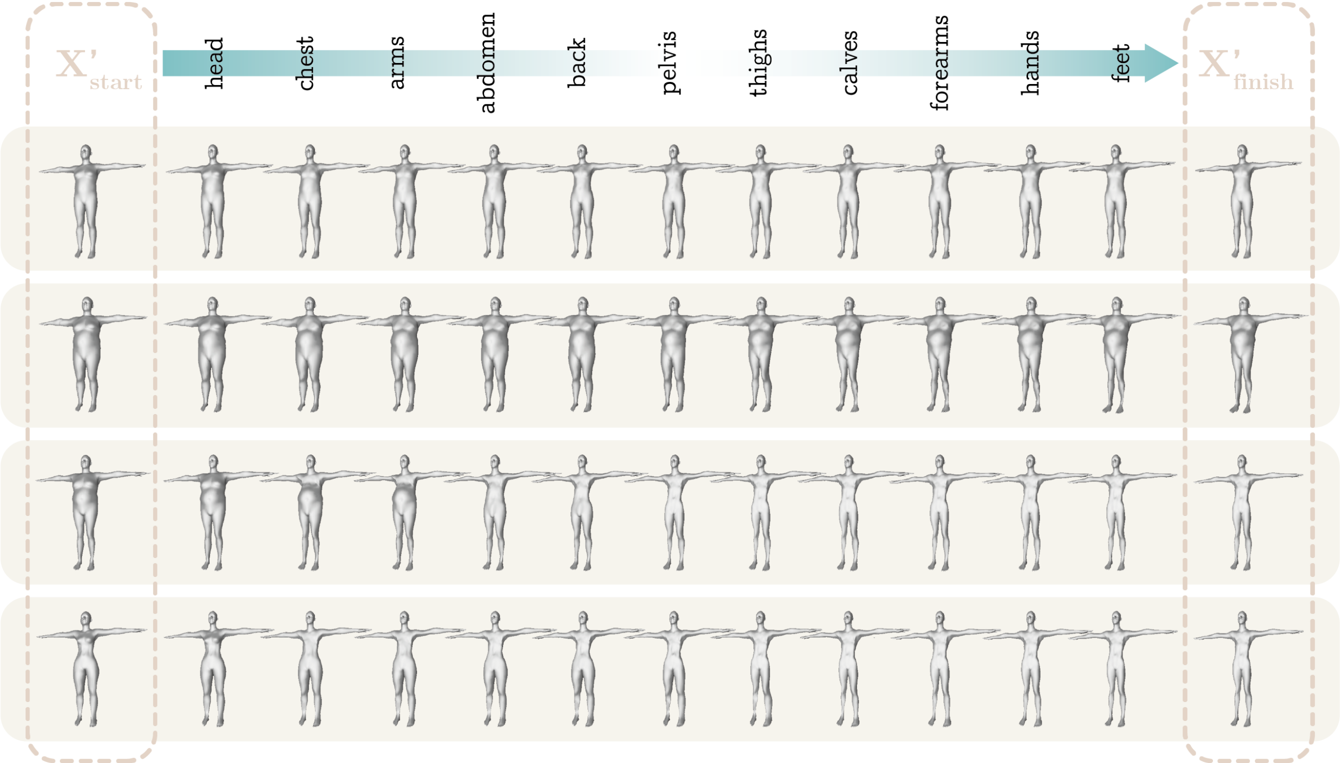

Figure 16: *Additional per-attribute latent replacements with LED-VAE trained on shapes from* STAR *(see Fig. 14).*

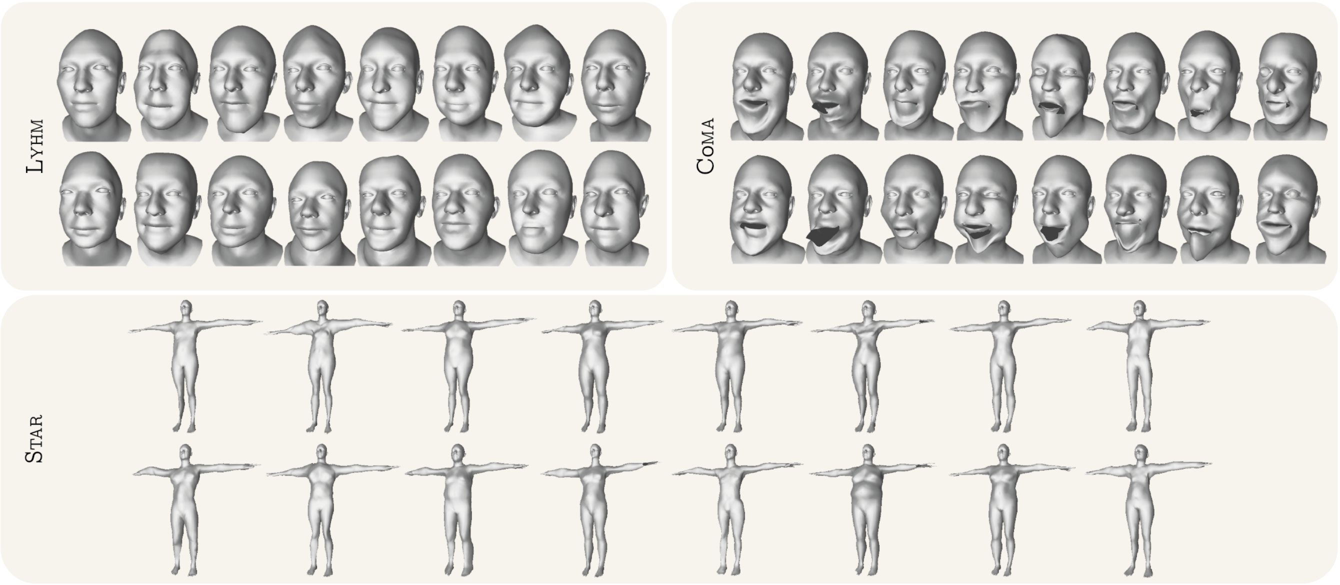

Figure 17: *Random samples generated by LED-VAE models trained on shapes from* LYHM, COMA, *and* STAR.

© 2023 The Author(s)
Computer Graphics Forum © 2023 The Eurographics Association and John Wiley & Sons Ltd.

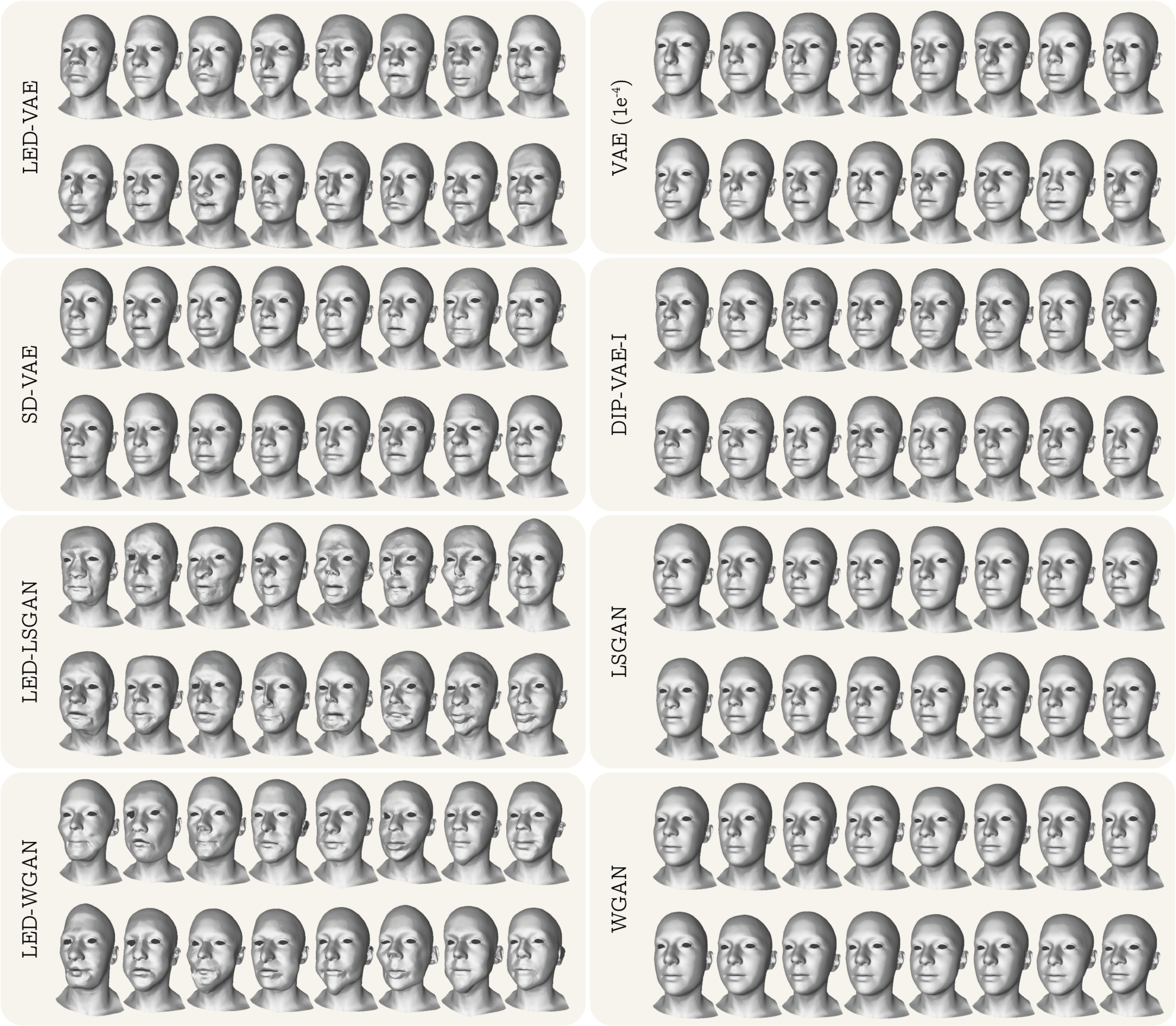

Figure 18: *Random samples generated by LED-VAE, SD-VAE, LED-LSGAN, LED-WGAN, VAE, DIP-VAE-I, LSGAN, WGAN. All models are trained on head shapes from* UHM.

© 2023 The Author(s)
Computer Graphics Forum © 2023 The Eurographics Association and John Wiley & Sons Ltd.

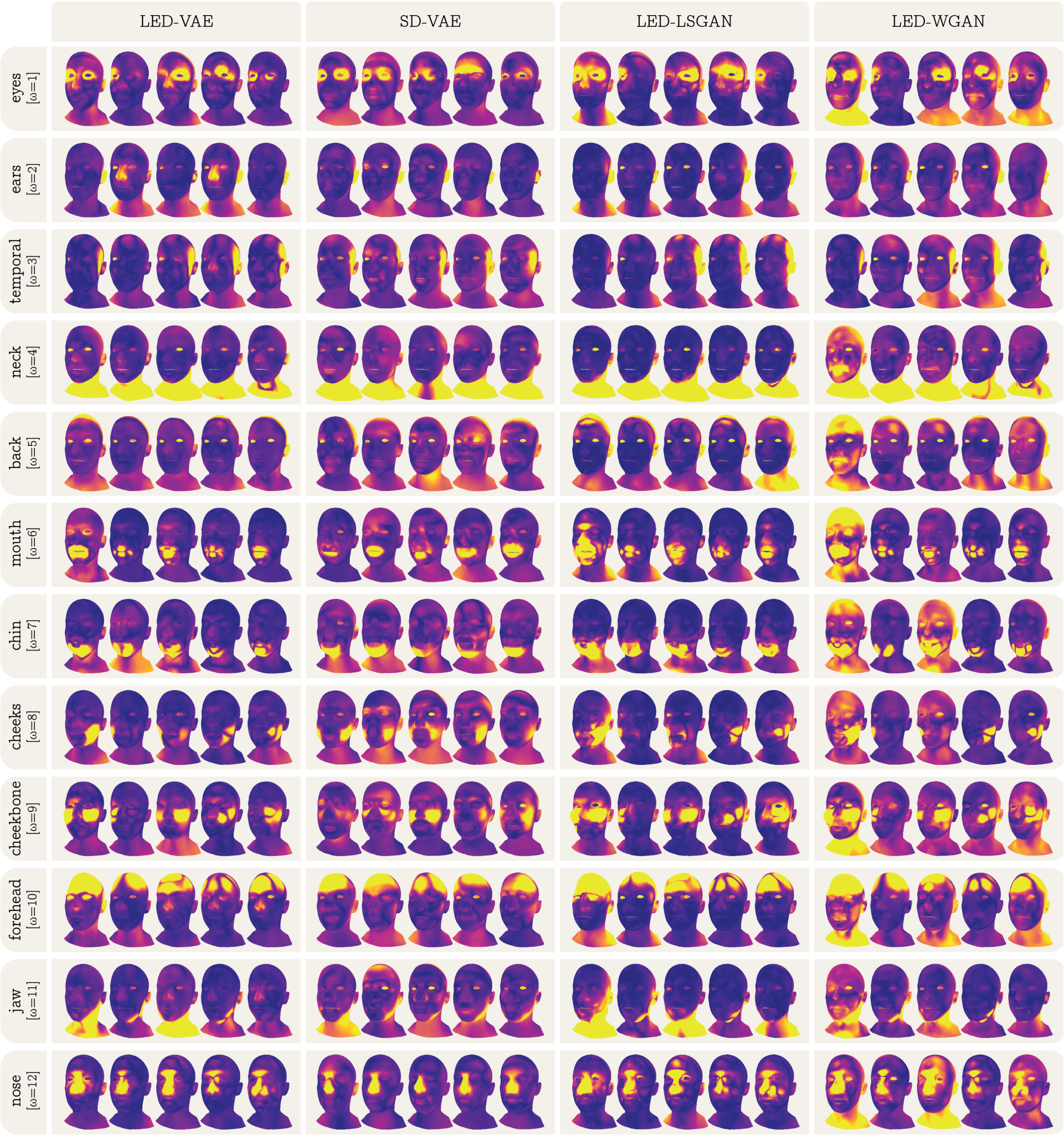

Figure 19: *Complete latent traversals grouped per-method along columns and per-attribute along rows. LED-VAE, SD-VAE, LED-LSGAN, and LED-WGAN are all trained on* UHM.

© 2023 The Author(s)
Computer Graphics Forum © 2023 The Eurographics Association and John Wiley & Sons Ltd.

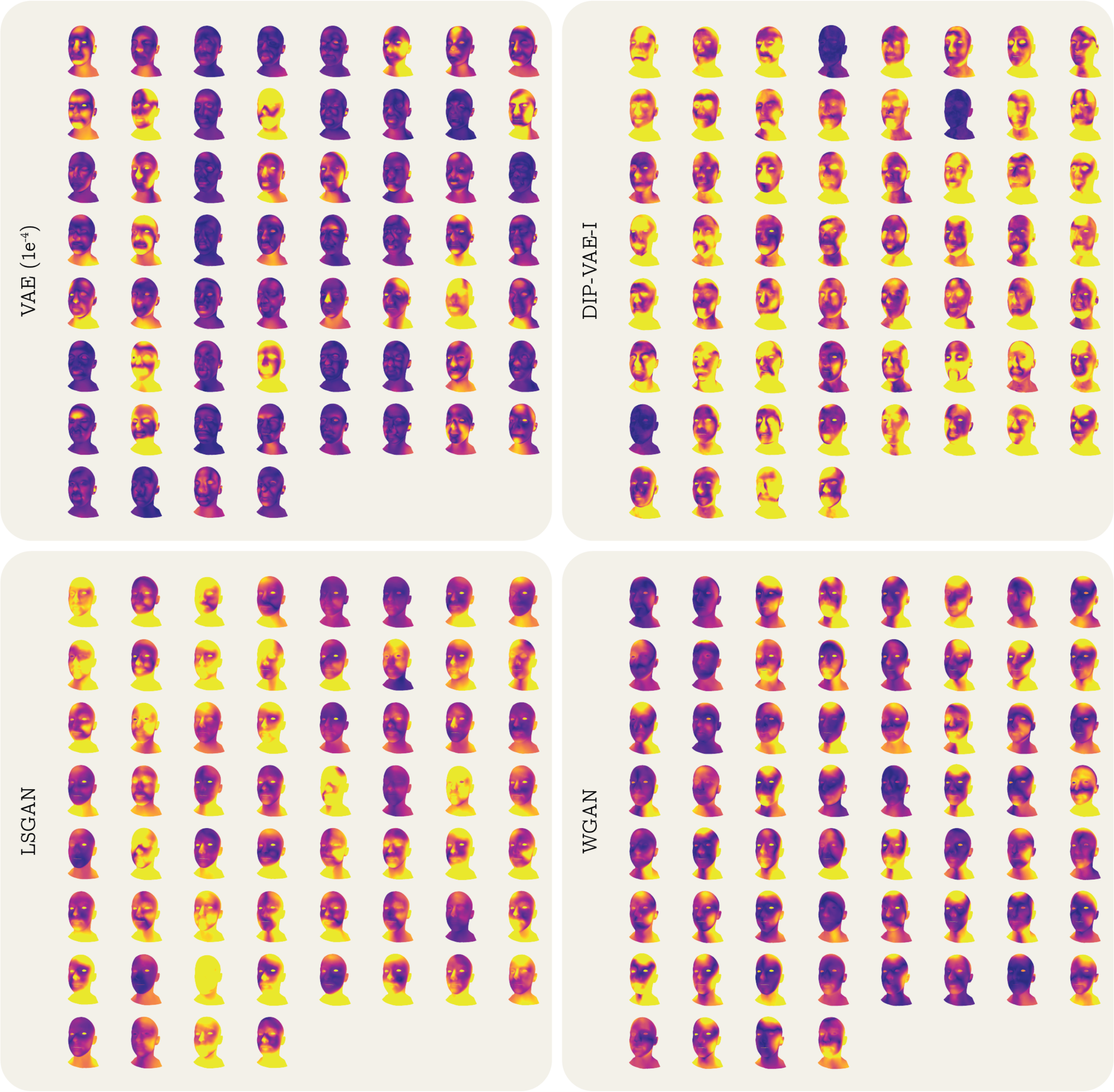

Figure 20: *Complete latent traversals of VAE, DIP-VAE-I, LSGAN, and WGAN trained on* UHM.

© 2023 The Author(s)
Computer Graphics Forum © 2023 The Eurographics Association and John Wiley & Sons Ltd.

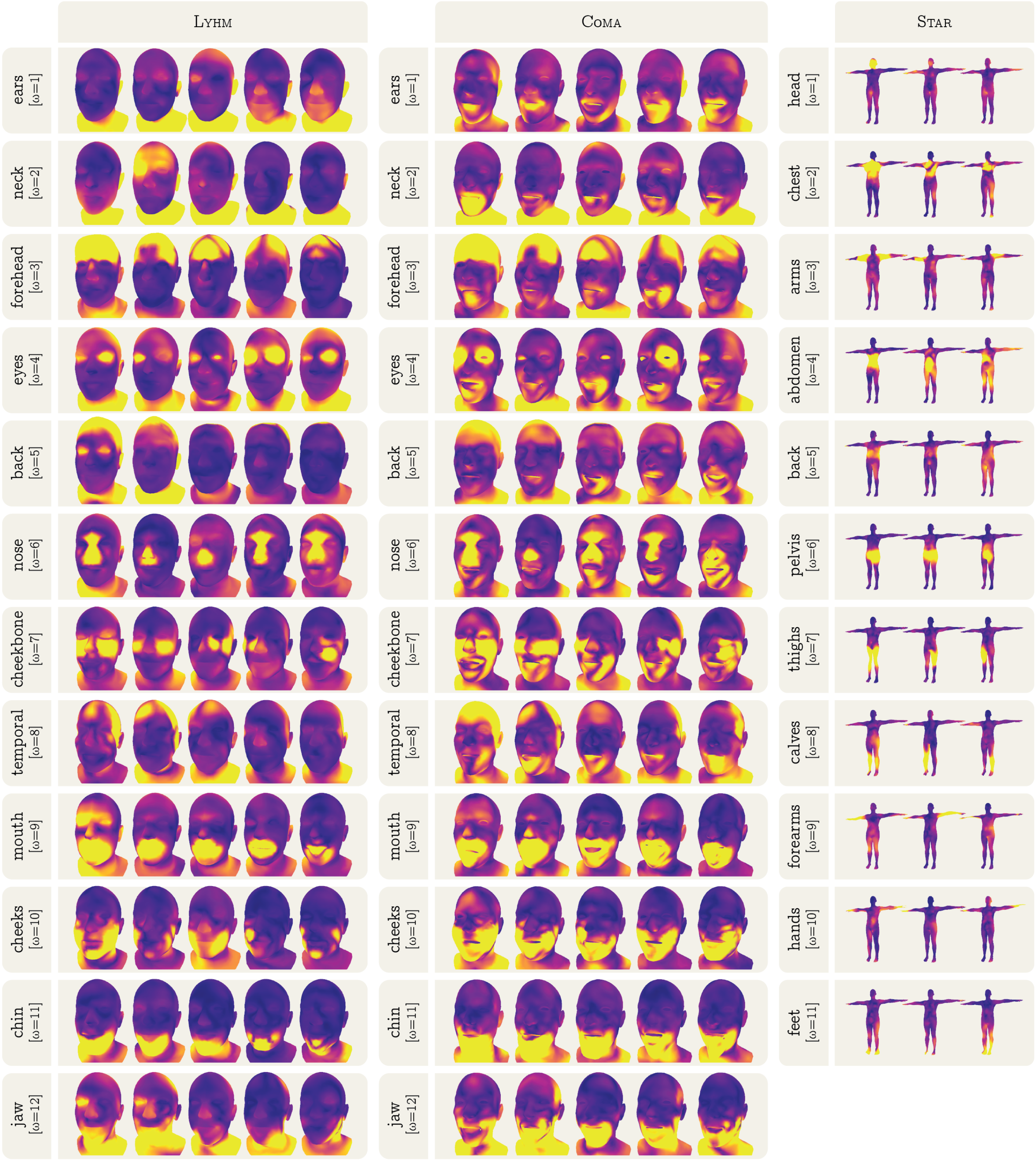

Figure 21: *Complete latent traversals of LED-VAE grouped per-dataset along columns and per-attribute along rows. The LED-VAE models are trained on shapes from* LYHM*,* COMA*, and* STAR*.*

© 2023 The Author(s)
Computer Graphics Forum © 2023 The Eurographics Association and John Wiley & Sons Ltd.
